# Consumer Acceptance of Alternative Proteins: Exploring Determinants of the Consumer Willingness to Buy in Germany

**DOI:** 10.3390/foods14142427

**Published:** 2025-07-09

**Authors:** Madita Amoneit, Leon Gellrich, Dagmara M. Weckowska

**Affiliations:** 1 School of Business and Economics, Freie Universität Berlin, 14195 Berlin, Germany; 2 Food4future (f4f), c/o Leibniz Institute of Vegetable and Ornamental Crops, 14979 Grossbeeren, Germany

**Keywords:** consumer acceptance, willingness to buy, alternative protein sources, protein transition, algae, crickets, jellyfish

## Abstract

In Western countries, a shift to a diet rich in proteins from diverse sources could aid the transition to more sustainable patterns of protein consumption and production, contributing to meet the future demand for protein from the growing population. The successful integration of alternative proteins into diets hinges, however, on consumer acceptance. Despite a plethora of acceptance studies on alternative proteins, comparative insights remain limited. To improve the fragmented understanding of the drivers and barriers of alternative protein acceptance, this study examines consumer willingness in Germany to buy food products containing proteins from three sources—algae, crickets and jellyfish—using the same methodological approach. The findings indicate that environmental consciousness strengthens the willingness to buy products based on all three protein sources while neophobia weakens it. In contrast, past meat consumption habits contribute positively to the acceptance of animal-origin alternative proteins, like crickets and jellyfish, but negatively to the acceptance of algae. The acceptance is also influenced by demographic factors. It is argued that strategies targeting these factors can enhance the acceptance of alternative protein sources such as algae, crickets and jellyfish. However, it is important to tailor the strategies to the determinants that influence the willingness to buy products from a particular protein source.

## 1. Introduction

A shift to a diet rich in proteins from diverse sources, as some argue, could enhance global food security [1] and contribute to transforming the dominant patterns of protein consumption and production in developed countries, which are associated with negative health and environmental impacts as well as animal welfare issues [2]. A variety of alternative proteins, such as those that are plant-based, insect-based, seafood-based, microbe-derived, and cultivated with cellular agriculture methods, among others, are widely discussed in the literature [3,4,5]. Diversification of protein intake in human diets is, however, challenging, as shown by numerous studies revealing barriers to consumer acceptance of alternative proteins [6,7,8]. Policy interventions could potentially help induce this dietary shift but the evidence needed to design effective interventions remains limited and fragmented.

Particularly problematic is the lack of comparative insights into acceptance across various protein sources. Past studies tend to focus on consumer acceptance of one specific protein source. The comparative insights are limited as the variety of measurements used in past studies makes cross-study comparisons difficult [9,10] and there are very few comparative studies examining more than one protein source [5,11]. Onwezen et al. (2021) underscore this research gap and call for future research that compares drivers and barriers across alternative protein sources [5].

The design of a comparative study, in particular, one on the choice of drivers and barriers relevant to multiple protein sources, is a difficult task for a number of reasons. First, past studies collectively reveal many factors that influence consumer acceptance of various protein sources, resulting in a long list of potentially relevant predictors. Second, predictors relevant to one protein source are often not important, or not tested, for another protein source. Additionally, even for a specific protein source, there are contradictory and inconclusive results about some predictors. This makes it difficult to specify a model that is relevant for multiple protein sources. Third, the stability of the effects found in past studies cannot be taken for granted. For example, what is perceived as novel changes over time and once new and unfamiliar foods may seem relatively familiar as time passes; hence, the influence of (un)familiarity is likely to wear off. Therefore, the past results have to be used carefully in hypothesising about patterns across multiple protein sources.

To generate comparative insights and address the aforementioned research gap, this study adopts an exploratory approach, based on stepwise regression, to identify the determinants of consumer acceptance of three protein sources—namely algae, crickets and jellyfish—focusing on consumers in Germany. These protein sources have been selected for three reasons. First, they are seen as possible options for diversification of protein intake. They have high protein content [12,13,14] and are seen as potential contributors to global food security [15,16,17,18]. They also require less land for cultivation, use fewer resources in production and emit fewer greenhouse gases compared to conventional meat [15,17,19]. Second, their consumption in Germany is uncommon and their potential for widespread adoption remains unclear [20]. Among them, algae are seen as the most desirable future food, followed by crickets and jellyfish [21]. Third, the selected protein sources differ in certain ways, allowing for interesting comparisons. Specifically, consumers in Germany are likely to be familiar with these protein sources to different degrees. Many types of algae have enjoyed long-term market presence in the European Union (EU) and are not classified as novel foods, but barriers to their acceptance persist [22,23]. Foods containing some insect-sourced proteins were recently authorised for animal and human consumption in the EU but consumer aversion remains one of the biggest challenges [19,24,25]. Jellyfish is currently classified as a novel food and is not allowed for sale in the EU, and only a few studies explore consumer attitudes and potential challenges [14,26].

This study examines the willingness to buy foods containing algae, crickets and jellyfish among 637 respondents in Germany. The study makes an empirical contribution by revealing the important drivers and barriers of consumer acceptance of selected protein sources among consumers in Germany. More importantly, thanks to a unified methodological approach, where the same determinants of consumer acceptance are analysed across three protein sources on the same sample of consumers, the quantitative results allow for a comparative analysis. The comparative narrative is based on hierarchical regression models that themselves do not permit direct statistical comparisons [27]. Nonetheless, the study makes a step towards closing the aforementioned research gap outlined by Onwezen et al. (2021) [5]. Notably, this study differs considerably from research on public perceptions of these three alternative protein sources, which examines the German consumers’ perceived possibility and probability of consuming these protein sources in the future [21].

The remainder of the paper is structured as follows: In the next section, the literature on the determinants of consumer acceptance of the specified alternative protein sources is discussed. Section 3 sets out the methodology. Section 4 presents the results, of which implications are then discussed in Section 5.

## 2. Theoretical Background

In this section, we review the consumer acceptance literature. We focus on studies that examine willingness to buy, primarily in Germany, Europe and other Western countries, where data are available.

### 2.1. Consumer Acceptance

Before the determinants of consumer acceptance can be discussed, the concept of acceptance itself needs to be defined. The term “consumer acceptance” enjoys widespread usage across various research fields and has been extensively reviewed within the food science sector [28,29,30] but there remains a lack of consensus regarding its essential components and a general definition. This overarching construct is employed with ambiguity across different disciplines, including profitability and sufficient user adoption in the form of purchase intention, as well as the absence of categorical rejection towards a product or technology, indicative of a general willingness to engage with it [31,32]. In their scoping review, Baker et al. (2022) define consumer acceptance based on various outcome measurements such as general acceptance, willingness to buy, willingness to pay, willingness to try, perceptions, consumption or purchase intention [9]. Wassmann et al. (2021) include the following measures as correlates of willingness to consume in their meta-analysis: willingness to adopt, willingness to eat, willingness to pay, and willingness to try [10]. However, achieving a concise yet comprehensive definition remains a desirable objective, and such a task exceeds the scope of this paper.

Nonetheless, it is crucial to define acceptance in each study to allow comparability. The existing literature on alternative protein sources centres around two main indicators, willingness to try [11,25,33,34] and willingness to buy [5,23,35,36,37,38,39,40], both of which are pivotal for gauging overall acceptance. Due to the lack of general guidance for selecting the indicators and given our specific interest in the potential of these three alternative protein sources to make substantial contributions to a change in diet, we have chosen to focus solely on willingness to buy, as it offers a direct insight into whether consumers are willing to make a purchase to include a specific protein source in their diets. Willingness to buy indicates the readiness to purchase food products of alternative protein sources (algae, crickets, jellyfish) in a grocery store, which goes beyond just the willingness to try [39]. In the following sections, we focus on the willingness to buy but also discuss the determinants of willingness to try and further correlates of consumer acceptance where previous studies on willingness to buy are lacking.

### 2.2. Neophobia

Food neophobia is one of the most investigated determinants of consumer acceptance, particularly in the context of food innovations. Food neophobia, defined as the aversion to and dislike of novel foods, is a trait that manifests with varying degrees of intensity among individuals [41]. Food neophobia negatively influenced consumer acceptance of algae-based foods [42] as well as post-tasting satisfaction with algae-based food products [43]. Food neophobia had a negative impact on the adoption intention of Spirulina-enhanced foods (a specific type of microalgae) in a sub-sample of Belgian consumers who know a lot about the latest food trends and like to eat but not in the sub-samples of vegetarian and sporty Belgian consumers [44]. Food neophobia also showed a negative effect on the willingness to try [33] and to eat or consume insects among German consumers [45,46,47]. A similar effect could be found for the willingness to adopt insect-based foods [25] and to buy them [39]. A cross-country comparative study (involving the Czech Republic, Germany, Finland, and Sweden) further revealed a negative effect on the willingness to buy insect-based foods [38], while the meta-analysis indicated a negative effect on the willingness to consume [10]. Furthermore, food neophobia negatively influenced the attitude towards consuming jellyfish [40].

When food is processed before sale, which is likely for the protein sources under study, a phobia against novel food technologies may also be relevant for consumer acceptance of such foods. Food technology neophobia is defined as the resistance to new or modern food technologies [48]. The effect on the consumer acceptance of algae-based products is not clear yet. Embling et al. (2022) did not find a significant effect [42]. However, the consumer willingness to consume insects [10,46] and readiness to adopt insects as meat substitutes [25] are influenced negatively by food technology neophobia. The relationship of food technology neophobia and consumer acceptance of jellyfish is still unclear.

To summarise, it is reasonable to assume that food neophobia is associated with low consumer acceptance of alternative protein sources. However, the evident lack of understanding of how food technology neophobia impacts consumer acceptance of algae and jellyfish necessitates further investigations.

### 2.3. Familiarity with New Foods

Familiarity with the concept of consuming algae and insects as well as past consumption experiences are also important determinants of consumer acceptance [5,25,47]. Perceiving algae-based food as tasty, edible and familiar significantly increased acceptance [42]. People who are familiar with eating insects are more likely to adopt insects in their diets [25] and be willing to consume insects [10]. In contrast, Lammers et al. (2019) have found no effect of familiarity on willingness to consume insect burgers [46]. To the best of the authors’ knowledge, no studies have specifically investigated the impact of familiarity on the consumer acceptance of jellyfish-based foods. However, there appears to be a general tendency for familiarity with the sea environment to positively influence the consumption of jellyfish [40].

Previous experiences with algae-based food have a positive impact on willingness to try and adopt algae into the diet [11]. Furthermore, experiences with eating insects lead to a higher willingness to try and adopt insect-based foods [11,33], to eat insect-based foods [39,45,46] and to buy insect-based food products [35,38]. However, a meta-analysis showed only a middle composite effect size of experience with willingness to consume insect-based foods (r = 0.35) [10]. Previous experiences with eating jellyfish lead to a higher willingness to try and adopt jellyfish in the diet [11].

Based on the previous findings, both familiarity and experience might have a positive effect on consumer acceptance of algae, crickets and jellyfish. However, some clarity is needed considering scholars that have found no or only a small effect, necessitating a unified research approach.

### 2.4. Food-Related Habits

Food-related lifestyle links individuals’ values with their eating behaviours, incorporating aspects such as food involvement and food innovativeness, as outlined in the food-related lifestyle framework by Brunsø et al. (2021) [49]. Food involvement refers to the extent to which individuals engage with and take an interest in different aspects of food [49]. Those with high food involvement regard food as an important part of their lives, dedicate considerable time and resources to it, and view it as essential for achieving personal goals [49,50]. Previous studies indicate that food involvement positively influences the intention to adopt Spirulina-enhanced foods among consumers who enjoy eating, though this effect is not observed among vegetarians, sporty individuals, or those well informed about the latest food trends [44]. Moreover, food involvement does not impact the willingness to try insect-based foods [51]. To the best of the authors’ knowledge, no studies have investigated the relationship between food involvement and the acceptance of jellyfish-based foods.

Another aspect of the food-related lifestyle is food innovativeness, which refers to the extent to which individuals adopt and integrate novel culinary practices and ingredients into their dietary habits [49]. In the absence of previous research on the relationship of food innovativeness and the consumer acceptance of alternative protein sources, Mazhar & Zilahy (2023) show that food-related lifestyles (including food involvement and food innovativeness) are moderately correlated to green food purchasing behaviour [52]. Furthermore, sensation seeking, a related construct, has a positive effect on willingness to consume insects in Germany [46]. Moreover, a meta-analysis revealed that food sensation and innovation seeking have a strong composite effect on willingness to consume insects (r = 0.29) [10].

A consistent research approach is required to bridge the knowledge gap about the direction and effect of food involvement and food innovativeness on consumer acceptance of algae, crickets and jellyfish.

The meat consumption of consumers is an important determinant of consumer acceptance of proteins from alternative sources [5]. A strong belief in the benefits of eating meat is associated with lower acceptance of algae-based foods among meat consumers [42]. Additionally, consumers with a high rate of weekly meat intake are less likely to choose snacks made out of algae (besides lentils/beans snacks, insect snacks, and hybrid meat snacks), with both the number of meat-eating days and preferred portion size serving as significant predictors of their dietary preferences [53]. Those following a vegetarian or vegan diet are less willing to eat insects [47]. Furthermore, consumers who perceive meat as nutritious, healthy, and strongly focusing on its taste are less inclined to adopt insects as meat substitutes [25]. However, consumers intending to reduce their meat consumption show higher readiness to adopt insects as meat substitutes [25]. Contrary to these findings, Lammers et al. (2019) reported that higher meat consumption is linked to higher acceptance of food containing invisible insects, although this effect disappears when other factors influencing the acceptance (e.g., sensation seeking, sustainability consciousness, food disgust) are considered [46]. A meta-analysis found only a small effect size between willingness to consume insects and meat consumption (r = 0.08) [10]. Schäufele et al. (2019) found no effect of meat consumption on the willingness to try insect-based food [33]. The relationship between meat consumption and consumer acceptance of jellyfish is still unclear.

In summary, it could be assumed that (high) meat consumption is associated with low consumer acceptance of algae-based protein. However, there is a clear knowledge gap regarding how meat consumption influences consumer acceptance of crickets and jellyfish. A consistent research approach is necessary to better understand its role.

### 2.5. Environmental and Health Consciousness

Promisingly, trends in Western societies towards healthier, natural and more environmentally friendly dietary habits facilitate the consumer acceptance of alternative protein sources [19,54]. Previous studies indicate that the individual degree of environmental consciousness (consumers who pay attention to the environmental impact of their food choices [25,47] has a significant effect on the consumer acceptance of algae, insects and plant-based meat alternatives [5]. The environmental friendliness and sustainability of algae are important reasons for eating them [55]. However, environmental consciousness has no significant effect on the adoption intention of Spirulina-enhanced foods [44]. Environmentally conscious consumers show higher readiness to adopt insects as meat substitutes than consumers with lower environmental consciousness [25]. However, some scholars have not found any impact of environmental consciousness on the willingness to consume insect-based foods [10,46,47]. The relationship between environmental consciousness and consumer acceptance of jellyfish is still unclear.

Even if a positive relationship between environmental consciousness and consumer acceptance of algae, crickets and jellyfish could be assumed, the current ambiguity regarding the role of environmental consciousness in consumer acceptance of algae, crickets and jellyfish demonstrates the need for more unified research.

Health consciousness describes consumers who pay attention to the health impacts of their food choices [25,56]. A preference for a healthy lifestyle can influence the acceptance of algae and insects as food sources [5]. Previous research found that health consciousness affects the intention to adopt Spirulina-enhanced foods [44]. However, Embling et al. (2022) found no significant effect of the acceptance of algae-based food products and health-related interest in foods [42]. The perception of healthiness significantly influences consumers’ willingness to purchase insect-based products [36] and to eat processed insect products in the future [47]. Other studies, however, found no significant effects of health consciousness on the readiness to adopt insects as a meat substitute [25] or on the willingness to consume insects [10]. How health consciousness correlates with consumer acceptance of jellyfish remains unclear.

The apparent gap in knowledge about the role of health consciousness in affecting consumer acceptance of algae, crickets and jellyfish highlights the need for a comprehensive research approach.

### 2.6. Demographic Characteristics

Previous findings on age show that younger consumers are more willing to try as well as to introduce algae into their diet [11]. However, some scholars have found no or unclear relationships between age and adoption intention [44], acceptance of algae-based food [42] and the choice of algae-based snacks [53]. A similar picture emerges for insects: younger consumers are more willing to eat and more ready to adopt insect-based foods [11,25,47] as well as more willing to buy insect-based (mealworm) food products [39]. Nevertheless, no or ambiguous effects have also been found for willingness to try, eat or consume insects [10,33,45,46,57] as well as for willingness to buy cricket-based protein powder before and after tasting [35]. The willingness to eat and adopt jellyfish-based food [11] and to buy food containing jellyfish [40] is higher among younger consumers. Overall, it can be summarised that consumer acceptance of all three alternative protein sources might decrease with age.

However, considering the studies found ambivalent or no age effects, the knowledge gap regarding the direction and effect of age on consumer acceptance of algae, crickets and jellyfish is still apparent. A unified research approach is needed to help clarify the role of age.

Gender appears to be an unclear determinant of consumer acceptance. Previous studies found that gender does not affect the willingness to try or adopt algae-based food [11], except for in the vegetarian sub-sample [44]. However, other studies found that women are less likely than men to purchase seaweed-based food products [58]. Furthermore, women tend to reject the consumption of insects more than men [10,11,25,33,39,45,47,57] and men are more willing to buy insect-based food products [38]. However, the influence of gender becomes negligible for the acceptance of invisible insects in food, contrasting with the acceptance of whole insects in food, where men exhibit a higher level of acceptance than women [47]. A similar pattern emerges when other factors are included (e.g., sensation seeking, environmental consciousness, food disgust) to explain consumer acceptance [46,47]. The willingness to try and adopt [11] as well as to buy jellyfish-based foods [40] is higher for men than for women.

Even though previous study findings suggest that men are more accepting of animal-origin alternative protein sources than women, the evident uncertainty about the influence of gender on consumer acceptance of algae, as well as crickets and jellyfish, underscores the necessity for a harmonised research approach.

In addition, the role of education in influencing consumer acceptance seems ambiguous. Consumers with increased levels of education are more likely to eat snacks made from algae [55]. Other scholars have found no effect of education on willingness to try insect-based foods [33], as well as willingness to buy mealworm-containing [39] and insect-based [38] food products. The level of education appears to have no effect on willingness to consume insect-based foods [10,45,47] or on the readiness to adopt insects as a meat substitute [25]. However, Lammers et al. (2019) found that a higher educational level is associated with higher acceptance of foods containing invisible insects, but this effect disappears when other determinants of acceptance (e.g., sensation seeking, sustainability consciousness, food disgust) are considered [46]. Consumers with higher levels of education tend to have a more positive attitude towards jellyfish consumption [40]. The lack of clarity about the direction and impact of education on consumer acceptance of algae, crickets and jellyfish highlights the need for a unified research approach.

Even if the direction and relevance of influence of demographic characteristics is not clear [5], they should be taken into account. This study focuses on the most common factors, age, gender and education, as they are the most investigated in other studies (e.g., Wassmann et al., 2021 [10]).

## 3. Materials and Methods

In the following section, we explain the methodological approach of the current study.

### 3.1. Data Collection

To assess the determinants of consumer acceptance regarding alternative protein sources, an online survey was conducted within the research project food4future, funded as part of the German Federal Ministry of Education and Research funding line Agricultural Systems of the Future. The data collection took place from 17 March 2023 until 26 October 2023 via the online survey platform SoSci Survey. During this time period, the questionnaire was distributed via multiple channels (social media, project website, press releases) and the reciprocal survey swapping platform ‘Survey Circle’ in order to reach a broad spectrum of German consumers.

### 3.2. Questionnaire

The survey utilised a structured questionnaire, which was modelled after a previous study employing a similar questionnaire [21]. However, the questionnaire was adjusted and refined to align with the purpose of the current investigation. At the beginning of the survey, participants were briefed on the overarching objectives of the food4future project and the study’s specific goals. They were informed that, upon completion, they would receive personalised feedback, which would include a comparison of their responses with the average answers from a previous study. Written informed consent was obtained from all participants. Subsequently, participants were queried about their demographics (age, gender, education). Following this, inquiries were made regarding their dietary habits (meat consumption) and general attitudes toward food (food neophobia, food technology neophobia, food involvement, food innovativeness, environmental consciousness, health consciousness). Then, algae, crickets and jellyfish were introduced using a short description text with a corresponding picture and participants were queried about their familiarity and past experiences with each alternative protein source and were tasked with evaluating their willingness to buy them. In order to minimise potential order effects, the three alternative protein sources—algae, crickets and jellyfish—were randomly presented in varying sequences for each participant. In general, within each set of multiple items addressing the same construct, the order of presentation was also randomised. Additionally, to maintain data quality, response times were recorded for every page completed by each participant.

### 3.3. Measurements

Participants were requested to provide demographic information, including age, gender and level of education. The latter was measured with nine answer options (according to ISCED-11 [59]) and it was converted based on the classification of the Federal Statistical Office of Germany into three categories: low, medium and high [60]. Meat consumption [61], familiarity ([47], original scale by Verbeke (2015) [25]) and past experiences ([47], original scale by Neves (2015) [62]) were measured with single items that consisted of multiple answer options to make it more convenient for participants to answer these questions, and afterwards, they were all converted into a dichotomous structure for all further analyses. Several scales were applied to measure the different determinants of consumer acceptance using five-point Likert scales, with low values indicating a low level of agreement with the given statement and high values indicating a high level of agreement. The original scale to measure food neophobia by Pliner & Hobden (1992) consisted of ten items [41]. However, we were hesitant to use item ten because the term ‘ethnic restaurant’ is not used in German language and needs to be either excluded or rephrased to, e.g., “places, where foods from other cultures are served” (see Siegrist et al., 2013, p. 295 [63]). Even though it seems to be common practice for other scholars, item exclusion is suboptimal and has an impact on comparability [25,64]. However, given the fact that rephrasing would also alter the original meaning of the item too much and the assumption that globalisation may have changed consumer responses to an item like item ten [65], we opted for exclusion in this case. Therefore, we also revised the item ‘Ethnic food looks too weird to eat’ into ‘Some food looks too weird to eat’. Furthermore, we decided to use only three out of thirteen items from the original food technology neophobia by Cox & Evans (2008) [48]. In the same way as Verbeke (2015) [25], we chose the three items from the first factor with the highest loadings, but excluded item four because it focused on ‘high-tech food products’, and since the alternative protein sources could be interpreted as such by participants, we wanted to avoid redundancy and focus instead solely on food technology with this measurement. According to Wendt & Weinrich (2023) [66], previous scholars also used different numbers of items from the scale. Food involvement and food innovativeness were measured on the scale proposed by Brunsø et al. (2021) [49]. Environmental consciousness was measured with five items [47] based on Meixner & Mörl von Pfalzen (2018) [67]. Health consciousness was assessed using three items according to Verbeke (2015) [25] who selected three items with the highest factor loadings from the eight-item General Health Interest scale by Roininen et al. (1999) [56]. All items were translated into German.

An internal consistency analysis (Cronbach’s alpha) was conducted revealing that Cronbach’s alpha for health consciousness is unsatisfactory (0.69) and dropping an item did not improve this. Thus, we excluded health consciousness from all further statistical analyses. The study variables can be found in Table 1. An overview of the questionnaire (including English and German items) as well as the item analyses can be found in Supplementary Files S1 and S2.

### 3.4. Data Analysis

All analyses were performed using R statistical software (v4.2.1 [68]). To investigate the factors influencing respondents’ willingness to buy food products containing the respective alternative protein source (0 = cannot imagine buying the product, 1 = can imagine buying the product), a binary logistic regression model was employed. The selection of the final model’s parameters was determined through stepwise regression, guided by the ‘Akaike Information Criterion’ (AIC) using the ‘MASS’ package in R (v7.3.57 [69]). The initial full model, prior to parameter selection, is represented as follows:

P(Y = Yes|X) = β0 + β1Age + β2Gender + β3Edu + β4MeatCons + β5Fam + β6Exp + β7FoodNeo + β8FoodTechNeo + β9FoodInv + β10FoodInno + β11Env(1)

Logistic regression results are often referred to as difficult to interpret [70,71]. In particular, the commonly reported odds ratios (ORs) are misinterpreted easily as risk ratios. Contrary to some practitioners’ beliefs, they are not comparable across different samples and models with different sets of explanatory variables [27]. Given the stepwise regression approach for each of the alternative protein sources, it is to be expected that the resulting models should differ at least somewhat regarding their set of explanatory variables. Thus, the marginal effects at the mean are calculated for each model using the ‘mfx’ package in R from Fernihough (2019) (v1.2.2) [72] and are also used for further interpretation. These effects represent the alteration in the likelihood of the outcome given a one-unit shift in the predictor or independent variable, keeping all other explanatory variables at their mean [73]. These should not be confused with the average marginal effects which represent the marginal effect averaged across all observation units, which usually does not differ much from the marginal effect at the mean [74]. Since researchers commonly report ORs, they will be calculated and reported as well. To evaluate the model’s fit, several pseudo-R^2^ measures were computed. Veall & Zimmermann (1994) [75] proposed that McKelvey and Zavoina’s R^2^ closely mirrors ordinary least squares R^2^, while Nagelkerke’s and McFadden’s R^2^ values tend to underestimate the ‘true’ R^2^. Consequently, McKelvey and Zavoina’s R^2^ values were utilised for result interpretation.

### 3.5. Data Quality Assessment

The inherent nature of online surveys being self-administered introduces a challenge in maintaining control over the data collection process. Inattentive participants may contribute responses that lack meaningful insight, potentially compromising the integrity of the dataset. This concern is particularly present when utilising a survey-swapping platform for participant recruitment. Random or careless responses have the potential to introduce noise into our dataset, possibly resulting in type I or type II errors. To mitigate these risks, both a priori and post hoc measures were employed to uphold data quality standards. The questionnaire layout was designed to be visually engaging, aimed at sustaining participant attention throughout the survey process. Additionally, each participant was incentivised with personalised feedback, serving as a motivational factor to encourage meaningful responses. Recognising that prolonged surveys often result in decreased attentiveness and quality of responses [76], we strategically structured the questionnaire to be completed within an average duration of 15 min. This approach aimed to optimise participant engagement and minimise the likelihood of inattentive or rushed responses. To identify potentially meaningless responses post hoc, we employed the measurement of response time per page, a reliable indicator for careless survey answering [77,78], although there is no consensus on how to identify suspicious response times [79]. Following Leiner’s (2019) recommendation, we utilised the relative speed index with a threshold of 2.0 to flag suspicious records [78]. Additionally, we incorporated Bowling et al.’s (2023) suggestion of a page time index, applying a two-second-per-item rule to identify excessively fast page completion times [80]. Participants were flagged as speeding if they completed pages at a rate faster than two seconds per item on at least three out of twelve relevant pages. Although the removal of speeding participants was already argued to be redundant [81], evidence suggests that inattentive responses contribute to diminished data quality and their exclusion can enhance the statistical power of a study [82]. Consequently, we opted to remove cases meeting either of the two criteria (relative speed index ≥ 2 or page time index ≥ 0.25) (more details can be found in the next section).

## 4. Results

### 4.1. Sample Characteristics

The sample for this study comprised 708 participants, with 637 individuals included in the analysis. Exclusion criteria were applied in cases where participants indicated a lack of serious response in the comments (n = 1), were underaged (n = 2), were flagged for excessive speeding (n = 35) or were not currently residing in Germany (n = 32). The median age of the participants was 31 years (M = 37.0, SD = 14.4, range 18–80 years). Among the participants, 379 (59.5%) identified as female, and 252 (39.6%) as male (0.9% identified as diverse or other and were recoded to ‘not applicable’ (NA) for methodological reasons). The majority (63.4%) held a college degree. The sample is biassed towards females as well as young and educated people in comparison to the general German population [83] (see also File S3). In terms of dietary characteristics, 49.2% reported consuming meat at least on a weekly basis in the past twelve months. A significant majority of participants (80.1%) expressed willingness to buy algae, in contrast to only a third (35%) who were willing to purchase crickets and just a quarter (26%) open to buying jellyfish. Algae also emerged as the most familiar and experienced food domain, with 97% of participants reporting familiarity and 83% having prior experience. While crickets are similarly familiar to most participants (95%), only 29% have actual experience with them. Jellyfish, on the other hand, are much less known, with only 30% of participants familiar with them as a food source, and a mere 8% having prior experience. A comprehensive overview of the sample characteristics is provided in File S1.

### 4.2. Stepwise Logistic Regression Result

The results of the stepwise logistic regression for each alternative protein source can be found in the following three tables, along with one predicted probability curve for an illustrative predictor. The predicted probability curves are derived using the ’ggpredict()’ function from the ‘ggeffects’ package in R from Lüdecke (v2.3.0) [84], while holding all other variables at their mean or reference levels. All predicted probability curves for each predictor included in the final models can be found in File S4. Further details regarding the stepwise selection process can be found in File S5.

**Figure 1:**
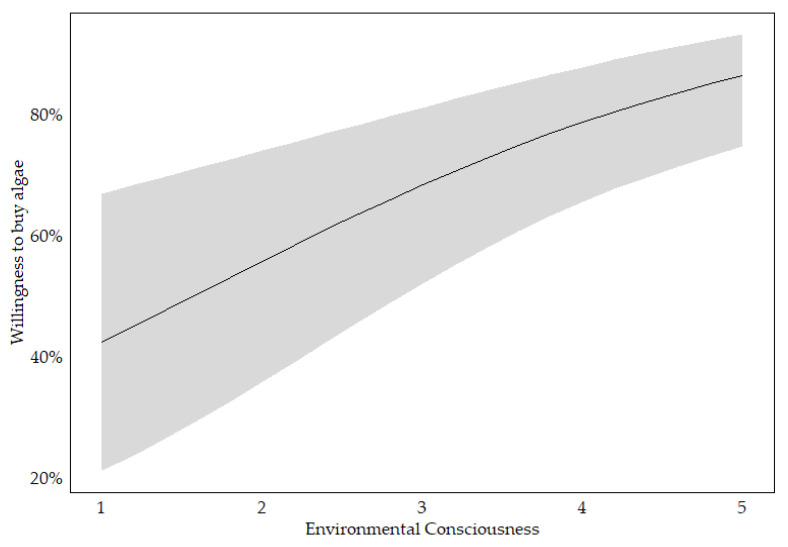
Predicted probability of willingness to buy algae across levels of environmental consciousness, based on the final logistic regression model; with 95% confidence interval (in grey).

During the stepwise selection process for algae, out of the initial eleven predictors, age, education and familiarity were removed from the model. The results for the final model for algae indicate significant associations between several independent variables and the binary dependent variable, willingness to buy, for this alternative protein source. Notably, experience exhibited the strongest effect, corresponding to a marginal effect of 24.7%. Additionally, environmental consciousness showed a significant positive association, with a marginal effect of 6.4% (see Figure 1). Meat consumption, food neophobia and food technology neophobia also exhibited significant but negative associations with willingness to buy algae, highlighting potential barriers to acceptance. Overall model fit statistics indicate moderate to good fit, with pseudo-R^2^ values ranging from 0.20 to 0.35, suggesting that the included variables explain a substantial portion of the variance in willingness-to-buy behaviour (see Table 2).

**Figure 2:**
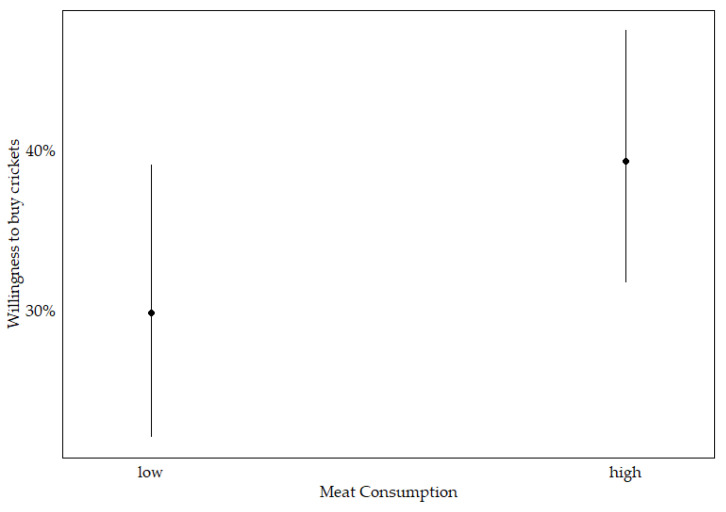
Predicted probability of willingness to buy crickets across levels of meat consumption, based on the final logistic regression model; with 95% confidence interval.

During the stepwise selection process for crickets, four predictors—education, familiarity, food involvement and food innovativeness—were excluded from the model out of the initial eleven predictors. The analysis reveals significant associations between several predictor variables and the binary dependent variable, willingness to buy, for cricket-based food products. Both age and environmental consciousness show a positive association with acceptance. Additionally, meat consumption, with a marginal effect of 9.2% (see Figure 2), and experience, with a marginal effect of 18.6%, are positively associated with willingness to buy. In contrast, gender is negatively associated with willingness to buy crickets, with a marginal effect of −12.1%. Both food neophobia and food technology neophobia also show negative associations, with food neophobia having the strongest marginal effect at 25.4%, indicating a substantial barrier to acceptance. The model’s pseudo-R^2^ values range from 0.16 to 0.28, suggesting moderate explanatory power (see Table 3).

**Figure 3:**
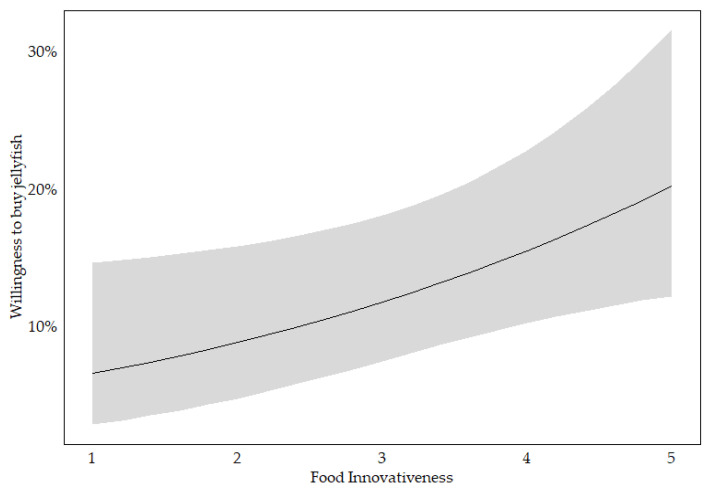
Predicted probability of willingness to buy jellyfish across levels of food innovativeness, based on the final logistic regression model; with 95% confidence interval (in grey).

The stepwise selection process for the jellyfish model resulted in the exclusion of age, education and food involvement. The analysis reveals significant associations between various predictor variables and the binary dependent variable, willingness to buy, for jellyfish-based food products. Gender is negatively associated with willingness to buy, showing a marginal effect of −6.6%. Jellyfish familiarity demonstrates the strongest positive association, with a marginal effect of 17.1%. Both food neophobia and food technology neophobia are negatively associated, indicating barriers to acceptance. Conversely, meat consumption, food innovativeness (see Figure 3) and environmental consciousness are positively associated with willingness to buy. The model’s pseudo-R^2^ values range from 0.17 to 0.31, reflecting moderate explanatory power (see Table 4).

The explanatory power of the models, as indicated by the pseudo-R^2^ values, is highest for algae, while the models for crickets and jellyfish show similar explanatory strength. Overall, the models explain between 28% and 35% of the variance in consumer acceptance (McKelvey and Zavoina’s R^2^: 0.28–0.35).

For all three alternative protein sources, each predictor variable has a VIF value lower than two in both the initial models and in the final models as well, which suggests that there is little to no multicollinearity among the predictor variables (see File S5).

## 5. Discussion

Shifting dietary habits towards diets rich in proteins from diverse sources is seen as one of the transition strategies towards more healthy diets, environmental sustainability [2] and enhanced food security [1]. However, the successful integration of alternative protein products in markets and diets is largely dependent on consumer acceptance [20]. Although a significant amount of research has been conducted on consumer acceptance of various protein sources, the comparative insights have been limited due to a lack of consistent measurement across studies (e.g., [9,10]) and a paucity of comparative studies focused on more than one protein source [5,11]. This exploratory study examined consumer acceptance across three different protein sources, namely algae, crickets and jellyfish, and generated some novel comparative insights. Thereby, the study makes an important step towards better understanding the differences in the acceptance of alternative protein sources.

### 5.1. Comparing Consumer Acceptance of Alternative Proteins in Germany and Beyond

The analysis across the three protein sources—algae, crickets and jellyfish—reveals both common and unique determinants of consumers’ willingness to buy these alternative protein sources. We find that food neophobia and food technology neophobia weaken consumer acceptance of all three alternative protein sources while environmental consciousness strengthens it. In contrast, the effects of experience and familiarity, food innovativeness, meat consumption, gender and age vary across the alternative protein sources.

Food neophobia, as one of the most studied factors influencing consumer acceptance, shows a consistently negative effect across all three alternative protein sources, with food-neophobic people showing lower consumer acceptance. Our results are consistent with previous findings for algae [42,43], crickets [10,25,33,38,39,45,46,47] and jellyfish [40]. In particular, food neophobia plays a notably important role among German consumers in shaping their acceptance to eat insect-based foods. This effect appears to be stronger in Central Europe (e.g., Germany, the Czech Republic) than in northern European countries (e.g., Finland, Sweden) [38]. Similarly, food technology neophobia negatively impacts the willingness to buy across the three alternative proteins, reinforcing the notion that technological and unfamiliar aspects of food processing may deter consumers. Our findings support previous findings on algae-based food [42] and insect-based food [10,46] that might be transferable to Western societies in general [25]. Additionally, our study is the first to examine neophobia’s effect on the acceptance of jellyfish-based food, where we also demonstrate a negative impact.

Environmental consciousness consistently shows a positive association with willingness to buy across all three alternative protein sources. This suggests that individuals who are more environmentally conscious are more likely to embrace alternative protein sources. Our findings are in line with those on crickets [25]. They also align with previous studies on algae in Australia [55] but differ from those of Moons et al. (2018), who reported no effect of environmental consciousness on the intention to adopt algae-enhanced food in a Belgian sample [44]. This may be explained by the fact that they distinguished between consumer segments (e.g., vegetarians, foodies) but did not assess environmental consciousness specifically in relation to food choices, as was performed in our measurement. Furthermore, our results contribute to a deeper understanding of the role of environmental consciousness in consumer acceptance of jellyfish-based foods as it was not investigated before.

Other factors show unique effects on the acceptance of each alternative protein source. Notably, familiarity with the respective protein source is a significant predictor only for jellyfish. This is in line with past findings among Italian consumers [40] and may be attributed to the fact that the idea of eating jellyfish is little known in Western countries including Germany and those already familiar with the idea are likely to be also highly interested in novel foods in general. In contrast, familiarity with eating algae and crickets is very high and does not discriminate between respondents. This highlights the importance of consumer familiarity and previous exposure in influencing acceptance of very novel protein sources [11].

Food innovativeness also determines the acceptance of jellyfish but not algae or crickets. It is possible that food innovativeness plays a more pronounced role in willingness to buy jellyfish because this is a highly novel food in the German context and hence is attractive to those who like to experiment with new foods. Our study is the first to report the positive effect of food innovativeness on consumer acceptance of jellyfish-based foods.

Experience with the respective protein source shows a strong positive effect on willingness to buy algae [11] and crickets [10,11,33,35,38,39,45,46] and is non-significant for jellyfish, contrary to previous findings [11]. Especially for Central Europe, including Germany, prior experiences with specific food products appear to play a more prominent role than in northern European countries [38].

Meat consumption plays a significant role in consumer acceptance across all three alternative proteins, but differs between algae- and animal-based protein sources. High meat consumption is negatively associated with the acceptance of algae-based foods, which has also been shown in previous studies [42,53]. In contrast, high meat consumption has a positive effect on the acceptance of animal-origin alternative protein sources such as crickets and jellyfish. This is also consistent with past findings showing that people who follow a vegan or vegetarian diet are less likely to accept eating insects [10,47]. However, our results contradict previous studies that found no effect of meat consumption on the acceptance of insect consumption based on a random sample of people walking through one German city [33]. Our study also sheds light on the so far unclear relationship between meat consumption and consumer acceptance of jellyfish, showing that high meat consumption positively correlates with the acceptance of jellyfish-based foods.

Identifying oneself as a woman shows a significant negative association with willingness to buy animal-origin protein sources such as crickets and jellyfish (and negative but non-significant for algae), as already shown in earlier studies investigating insects [10,11,25,33,39,45,47,57] and jellyfish [11,40]. Our findings are in line with previous studies that found no gender effect on consumer acceptance of algae-based foods [11,44]. This contrasts with earlier findings from Li et al. (2021), who found that men are more likely to purchase seaweed-based products than women in an U.S.-American sample [58].

Furthermore, age demonstrates a significant positive association only with willingness to buy crickets, suggesting that older individuals in Germany may be more receptive to trying crickets, contradicting previous studies that found younger consumers show higher consumer acceptance [11,25,39,47]. Orsi et al. (2019) also investigated a German sample similar to ours, but they measured the willingness to eat insects [47], whereas our study focused on the willingness to buy cricket-based foods. This difference highlights the need for further research to better understand the contradictory effects of age among German consumers. However, age does not appear to significantly impact willingness to buy the other two protein sources (similarly to [42,44,53]). Contrary to our findings, age effects on consumer acceptance of algae-based [11] and jellyfish-based foods [11,40] have been found in previous studies and no or ambiguous age effects on acceptance for insect consumption [10,33,35,45,46,57].

Even though education [40,55] and food involvement [44] were significant predictors in other studies with Australian, Italian, and Belgian samples, they did not emerge as significant predictors of willingness to buy in this study, illustrating cross-country differences. The unique role of Germany in this context warrants further investigation to better understand the individual and contextual factors shaping consumer acceptance of alternative protein sources.

### 5.2. Practical Implications

The following practical implications can be drawn: for all three protein sources, strategies changing the perceived newness of the protein source could weaken the negative effects of neophobia, while strategies strengthening the environmental performance of the food products and environmental consciousness of consumers could enhance consumer acceptance. Acceptance of jellyfish could also be strengthened by strategies raising awareness of this food option and enabling culinary experiments. However, strategies to reduce the novelty of alternative protein sources may undermine the positive influence of food innovativeness on consumer acceptance of jellyfish-based foods, and such timing of such strategies should be carefully considered.

### 5.3. Limitations and Future Research

The study’s design and the sample have affected the results in the following ways:

First, the consumer acceptance of alternative proteins among respondents in our sample, in which young, female and highly educated people were over-represented, may have been more positive than in the general population in Germany as these groups tend to be environmentally conscious [85]. A similar effect may have been due to the high proportion of people who eat meat less than once a week (49.3%). Future studies based on representative samples are needed to address the potential overestimation of consumer acceptance of the three alternative protein sources due to our skewed sample.

Second, we follow an exploratory approach aiming to fill in the gap of consumer acceptance research in comparing across various drivers and different alternative proteins [5]. Thereby, our findings and indicated conclusions should be investigated further in experimental study designs to examine determinants’ causal inferences that we cannot deduce with our study design. In particular, the role of health consciousness remains unclear due to the lack of reliable measurement in our study.

Third, this study did not separate macro- from microalgae, consistent with Mellor et al. (2022) [23]. However, since these species differ significantly in their metabolomic profiles [86], this may weaken the robustness of our results. Future research should consider examining each type independently.

Fourth, some of the scales employed were abbreviated versions that had not been previously validated. Additionally, the translation of these scales into German may have further impacted their validity. This is particularly evident in the health consciousness scale, which, due to translation and the use of only three out of eight original items, exhibited poor internal consistency and was thus excluded. Similarly, the scales for food neophobia and food technology neophobia were also abbreviated with only nine out of ten items and three out of thirteen items, respectively. These modifications may have affected the reliability and validity of our measurements. However, these trade-offs were necessary to ensure a manageable survey length and minimise respondent fatigue, given the study’s broad scope and general population sample. To mitigate potential limitations, we undertook several steps. We systematically selected items based on strong factor loadings from the original scales, in line with previous research (e.g., Verbeke, 2015 [25]), and conducted a comprehensive item analysis to assess internal consistency and item performance. Items that did not meet acceptable quality standards were excluded (e.g., health consciousness). Additionally, we piloted the survey with a small sample (n = 25) to assess the comprehensibility and usability of the translated items. While this pilot was not designed for full psychometric validation, it ensured that the materials functioned as intended. Taken together, these steps reflect our effort to ensure measurement quality despite constraints, and underscore the need for the development and validation of concise, psychometrically robust German-language scales in future research.

Fifth, while our stepwise regression approach has provided valuable insights, it does have potential limitations. Stepwise regression can be prone to overfitting, particularly in the absence of cross-validation, which might affect the generalisability of our models to other samples. Additionally, this method prioritises statistical fit, potentially overlooking variables with theoretical significance. Given that our predictors were derived from literature that did not simultaneously account for all variables, we accepted the risk of excluding theoretically relevant predictors in favour of focusing on statistically significant ones. Although penalised regression methods such as LASSO or Ridge could be beneficial for selecting determinants while mitigating overfitting, we opted not to use these techniques [87,88]. This decision was based on the exploratory nature of our study and the goal of understanding potential predictors without imposing additional constraints that penalised methods introduce. We also considered hierarchical regression but dismissed it due to the lack of a robust theoretical framework to support a well-founded hierarchical structure for our predictors.

Sixth, the moderate pseudo-R^2^ values in our study suggest that, while the models explain some of the variance in the willingness to buy, there remains a significant amount of unexplained variability. This might indicate the presence of important predictors or factors that were not included in the study. Also, our models may not fully capture the complexity of consumer acceptance and might be too simplified, missing important interactions or non-linear relationships.

Seventh, the dependent variable in this study measures the willingness to buy rather than actual purchasing behaviour. Intention is a predictor of behaviour but does not always translate directly into actual purchasing decisions. The study also did not specify the product or form in which the alternative protein sources would be offered for purchase. Participants might be open to consuming products containing cricket or jellyfish powder but may be less willing to purchase or consume the animal in a rather unprocessed form (e.g., [33,45,89]). This lack of specificity regarding the food products may have either led to an overestimation of willingness to buy, as participants might have imagined more familiar or acceptable product formats, or led to an underestimation, particularly in the case of jellyfish, as participants may have struggled to envision how such food products containing jellyfish could be prepared or consumed. Further research is needed to better understand actual purchasing behaviour and the influence of food product types on alternative protein sources.

## 6. Conclusions

The study aims to address the gap in the current consumer acceptance literature that requires comparative analysis of alternative proteins to provide a more comprehensive understanding of consumer acceptance of alternative protein sources. By investigating algae, crickets and jellyfish as alternative protein sources, we have selected promising proteins that could contribute to global food security and have contrasted algae-based (algae) and animal-origin (crickets, jellyfish) alternatives, considering both more established protein alternatives (algae), new options (crickets) and rather unknown alternatives (jellyfish). The novelty of this study lies in its unified methodological approach, leveraging the same determinants of consumer acceptance, a consistent sample and a harmonised statistical approach to facilitate meaningful narrative comparability. The study also contributes to deepening the understanding of consumer acceptance of jellyfish.

## Figures and Tables

**Table 1:** Overview of study variables.

Variable	Description	Mean (SD)	Cronbach’s Alpha
Age	one continuous item (year)	37.01 (14.39)	-
Education (Edu)	1 = low, 2 = medium, 3 = high	2.54 (0.66)	-
Gender	0 = male, 1 = female	0.60	-
Meat Consumption (MeatCons)	0 = less than weekly, 1 = weekly or daily	0.51	-
Familiarity (Fam) [algae/crickets/jellyfish]	0 = not familiar, 1 = familiar	0.97/0.95/0.30	-
Experience (Exp) [algae/crickets/jellyfish]	0 = no prior experience, 1 = prior experience	0.83/0.29/0.08	-
Food Neophobia (FoodNeo)	nine items with 5-point Likert scale (1 = low to 5 = high)	2.59 (0.66)	0.82
Food Technology Neophobia (FoodTechNeo)	three items with 5-point Likert scale (1 = low to 5 = high)	2.47 (0.78)	0.74
Food Involvement (FoodInv)	five items with 5-point Likert scale (1 = low to 5 = high)	4.16 (0.63)	0.80
Food Innovativeness (FoodInno)	six items with 5-point Likert scale (1 = low to 5 = high)	3.58 (0.9)	0.90
Environmental Consciousness (Env)	five items with 5-point Likert scale (1 = low to 5 = high)	3.68 (0.81)	0.89
Health Consciousness (Heal)	three items with 5-point Likert scale (1 = low to 5 = high)	3.54 (0.78)	0.69
Willingness to buy (WTB) [algae/crickets/jellyfish]	0 = cannot imagine buying the product, 1 = can imagine buying the product	0.80/0.36/0.23	-

**Table 2:** Final parameters of the binary logistic regression model after the stepwise selection process for algae.

Variable	β	Standard Error	*z*-Value	*p*-Value	Odds Ratio	Marginal Effect
(Intercept)	3.216	1.211	2.655	<0.01	-	-
Gender	−0.438	0.254	−1.725	ns	0.645	−5.1%
MeatCons	−0.560	0.258	−2.173	<0.05	0.571	−6.7%
Exp	1.491	0.267	5.574	<0.001	4.440	24.7%
FoodNeo	−0.784	0.222	−3.530	<0.001	0.456	−9.4%
FoodTechNeo	−0.573	0.159	−3.604	<0.001	0.564	−6.8%
FoodInv	−0.383	0.219	−1.746	ns	0.682	−4.6%
FoodInno	0.273	0.173	1.579	ns	1.314	3.3%
Env	0.537	0.147	3.657	<0.001	1.711	6.4%

Note. Binary dependent variable: willingness to buy (0 = no and 1 = yes); pseudo-R^2^: Cox and Snell’s R^2^ = 0.20, Nagelkerke’s R^2^ = 0.32, McFadden’s R^2^ = 0.23, McKelvey and Zavoina’s R^2^ = 0.35; ns = not significant.

**Table 3:** Final parameters of the binary logistic regression model after the stepwise selection process for crickets.

Variable	β	Standard Error	*z*-Value	*p*-Value	Odds Ratio	Marginal Effect
(Intercept)	1.352	0.686	1.969	<0.05	-	-
Age	0.016	0.007	2.346	<0.05	3.864	0.35%
Gender	−0.543	0.194	−2.803	<0.01	1.016	−12.1%
MeatCons	0.421	0.204	2.062	<0.05	0.581	9.2%
Exp	0.813	0.203	4.015	<0.001	1.524	18.6%
FoodNeo	−1.159	0.173	−6.688	<0.001	2.255	−25.4%
FoodTechNeo	−0.300	0.130	−2.311	<0.05	0.314	−6.6%
Env	0.255	0.128	1.992	<0.05	0.741	5.6%

Note. Binary dependent variable: willingness to buy (0 = no and 1 = yes); pseudo-R^2^: Cox and Snell’s R^2^ = 0.19, Nagelkerke’s R^2^ = 0.26, McFadden’s R^2^ = 0.16, McKelvey and Zavoina’s R^2^ = 0.28.

**Table 4:** Final parameters of the binary logistic regression model after the stepwise selection process for jellyfish.

Variable	β	Standard Error	*z*-Value	*p*-Value	Odds Ratio	Marginal Effect
(Intercept)	−0.925	1.014	−0.912	ns	0.397	-
Gender	−0.430	0.217	−1.983	<0.05	0.651	−6.6%
MeatCons	0.515	0.230	2.244	<0.05	1.674	7.7%
Fam	1.008	0.224	4.508	<0.001	2.740	17.1%
Exp	0.599	0.365	1.642	ns	1.821	10.5%
FoodNeo	−0.941	0.210	−4.494	<0.001	0.390	−14.1%
FoodTechNeo	−0.315	0.148	−2.121	<0.05	0.730	−4.7%
FoodInno	0.322	0.145	2.218	<0.05	1.380	4.8%
Env	0.314	0.152	2.072	<0.05	1.369	4.7%

Note. Binary dependent variable: willingness to buy (0 = no and 1 = yes); pseudo-R^2^: Cox and Snell’s R^2^ = 0.17, Nagelkerke’s R^2^ = 0.26, McFadden’s R^2^ = 0.18, McKelvey and Zavoina’s R^2^ = 0.31; ns = not significant.

## Data Availability

The data presented in this study are available on request from the corresponding author due to privacy restrictions.

## Supplementary Materials

The following supporting information can be downloaded at https://www.mdpi.com/article/10.3390/foods14142427/s1, File S1: Survey items; File S2: Item analyses; File S3: Comparison between German population vs. sample; File S4: Predicted probability curves; File S5: Stepwise logistic regression.
