# Supplementary material for: Consumer Acceptance of Alternative Proteins: Exploring Determinants of the Consumer Willingness to Buy in Germany
Source: Foods. 2025 Jul 9;14(14):2427. doi: 10.3390/foods14142427 (PMC12294508; doi:10.3390/foods14142427)
Supplement: Supplementary file 1 [file foods-14-02427-s001.zip › foods-3686952-supplementary.pdf]

Supplementary Materials

File S1: Survey items

Table S1. Survey items.

Variable (reference)	Item (English)	Item (German)	Answer options (English)	Answer options (German)	No. of participants (%)	Used categories for analyses
**Age**	How old are you?	Wie alt bist Du?	continuous (year)	18 - 29: 310 (48.7)<br>30 - 39: 112 (17.6)<br>40 - 49: 63 (9.9)<br>50 - 59: 104 (16.3)<br>60 - 69: 41 (6.4)<br>70 - 79: 7 (1.1)<br>>80: 0<br>NA: 0	continuous (year)	
**Gender**	Which gender do you assign yourself?	Welchem Geschlecht ordnest Du Dich zu?	male	männlich	252 (39.6)	0 = male
			female	weiblich	379 (59.5)	1 = female
			diverse	divers	6 (0.9)	NA
			missing values	0	NA	
**Education** [60]	What is your highest level of education?	Was ist Dein höchster Bildungsabschluss?	still a student	noch Schüler/in	4 (0.6)	1 = low
			finished school without a degree	Schule beendet ohne Abschluss	2 (0.3)	1 = low
			Primary school	Hauptschulabschluss oder Volksschulabschluss	7 (1.1)	1 = low
			Secondary school	Realschulabschluss (Mittlere Reife) oder gleichwertiger Abschluss	44 (6.9)	1 = low
			Polytechnic secondary school	Abschluss Polytechnische Oberschule 10. Klasse (vor 1965: 8. Klasse)	4 (0.6)	1 = low
			Technical college	Fachhochschulreife (Abschluss einer Fachoberschule)	14 (2.2)	2= medium
			A-level	Abitur, allgemeine oder fachgebundene Hochschulreife (Gymnasium bzw. EOS)	158 (24.8)	2= medium
			University of Applied Sciences or university	Fachhochschul- oder Hochschulabschluss	404 (63.4)	3 = high
			other educational qualification, namely:	anderer Schulabschluss, und zwar:	0	NA (all were recoded into the existing categories)
			missing value	0	NA	

**Meat Consumption** [61]	How often do you consume meat and/or meat products in the last 12 months?	Wie oft hast du in den letzten 12 Monaten Fleisch und/oder Fleischerzeugnisse verzehrt?	never	nie	144 (22.6)	0 = less than weekly
			less than once a month	weniger als einmal im Monat	49 (7.7)	0 = less than weekly
			one to three times a month	ein-bis dreimal im Monat	121 (19.0)	0 = less than weekly
			one to four times a week	ein-bis viermal in der Woche	248 (38.9)	1 = weekly or daily
			almost daily/daily	fast täglich/täglich	75 (11.8)	1 = weekly or daily
			missing value	0	NA	
**Familiarity** [47], original scale by [25]	Have you ever heard of eating algae/crickets/jellyfish and know what it implies?	Hast Du schon jemals zuvor von Algen/Grillen/Quallen als Nahrungsquelle gehört?	Yes, I have heard of the eating of algae/crickets/jellyfish and I know what it implies.	Ja, ich habe von Algen/Grillen/Quallen als Nahrungsquelle gehört und kann mir was darunter vorstellen.	528 (82.9) / 524 (82.3) / 97 (15.2)	1 = familiar
			Yes, I have heard of the eating of algae/crickets/jellyfish but actually don't know what it implies.	Ja, ich habe von Algen/Grillen/Quallen als Nahrungsquelle gehört, kann mir aber nichts darunter vorstellen.	85 (13.3) / 81 (12.7) / 93 (14.6)	1 = familiar
			No, I have never heard of the eating of algae/crickets/jellyfish.	Nein, ich habe noch nie von Algen/Grillen/Quallen als Nahrungsquelle gehört.	22 (3.5) / 31 (4.9) / 447 (70.2)	0 = not familiar
			missing value	2 (0.3) / 1 (0.2) / 0	NA	
**Experience** [47], original scale by [62]	Have you ever tried algae/crickets/jellyfish? (e.g., as a food product, as an ingredient in food)	Hast Du schon mal Algen/Grillen/Quallen gegessen oder probiert? (z.B. als Lebensmittelprodukt, als Inhaltsstoff in Lebensmitteln)	I eat algae/crickets/jellyfish regularly.	Ja, ich esse regelmäßig Algen/Grillen/Quallen.	61 (9.6) / 1 (0.2) / 0	1 = prior experience
			I have tried algae/crickets/jellyfish on a few occasions.	Ja, ich habe Algen/Grillen/Quallen öfters gegessen oder probiert.	282 (44.3) / 38 (6.0) / 12 (1.9)	1 = prior experience
			I have tried algae/crickets/jellyfish on a single occasion.	Ja, ich habe Algen/Grillen/Quallen einmal gegessen oder probiert.	165 (25.9) / 132 (20.7) / 16 (2.5)	1 = prior experience
			I have never tried algae/crickets/jellyfish in any form.	Nein, ich habe noch nie Algen/Grillen/Quallen gegessen oder probiert.	105 (16.5) / 450 (70.6) / 586 (92.0)	0 = no prior experience
			I do not know.	Ich weiß nicht.	22 (3.5) / 15 (2.4) / 23 (3.6)	NA
			missing value	2 (0.3) / 1 (0.2) / 0	NA	
**Food Neophobia** [41]	I am constantly sampling new and different foods. (R)	Ich probiere ständig neue und andere Lebensmittel aus. (R)	5-point Likert scale (1 = strongly disagree, 2 = disagree, 3 = partly,	5-point Likert scale (1 = stimme ganz und gar nicht zu, 2 = stimme nicht zu, 3 = teils, teils, 4 = stimme	1 = 59 (9.3), 2 = 187 (29.4), 3 = 246 (38.7), 4 = 107 (16.8), 5	all values were averaged into a mean score

		partly, 4 = agree, 5 = strongly agree)	zu, 5 = stimme voll und ganz zu)	= 36 (5.7), NA = 2 (0.3)
I don't trust new foods.	Ich traue neuartigen Lebensmitteln nicht.			1 = 117 (18.4), 2 = 279 (43.8), 3 = 184 (28.9), 4 = 45 (7.1), 5 = 10 (1.6), NA = 2 (0.3)
I like foods from different countries. (R)	Ich mag Lebensmittel aus verschiedenen Ländern. (R)			1 = 241 (37.9), 2 = 297 (46.7), 3 = 79 (12.4), 4 = 15 (2.4), 5 = 3 (0.5), NA = 2 (0.3)
If I don't know what is in a food, I won't try it.	Wenn ich nicht weiß, was in einem Lebensmittel enthalten ist, esse ich es nicht.			1 = 45 (7.1), 2 = 148 (23.2), 3 = 212 (33.3), 4 = 159 (25.0), 5 = 71 (11.2), NA = 2 (0.3)
At dinner parties, I will try a new food. (R)	Bei Dinnerpartys probiere ich neues Essen aus. (R)			1 = 157 (24.6), 2 = 282 (44.1), 3 = 130 (20.3), 4 = 50 (7.8), 5 = 16 (2.5), NA = 2 (0.3)
Some food looks too weird to eat.	Manche Lebensmittel sehen zu komisch aus, um sie zu essen.			1 = 60 (9.4), 2 = 143 (22.5), 3 = 196 (30.9), 4 = 184 (29.0), 5 = 49 (7.7), NA = 5 (0.8)
I am afraid to eat things I have never had before.	Ich habe Angst davor, Dinge zu essen, die ich noch nie gegessen habe.			1 = 162 (25.4), 2 = 277 (43.4), 3 = 143 (22.4), 4 = 41 (6.4), 5 = 12 (1.9), NA = 2 (0.3)
I am very particular about the foods I will eat.	Ich bin sehr wählerisch bei den Lebensmitteln, die ich esse.			1 = 48 (7.5), 2 = 137 (21.5), 3 = 220 (34.5), 4 = 165 (25.8), 5 = 65 (10.2), NA = 2 (0.3)
I will eat almost anything. (R)	Ich esse fast alles. (R)			1 = 92 (14.5), 2 = 207 (32.6), 3 = 132 (20.8), 4 = 145 (22.9), 5 = 59 (9.3), NA = 2 (0.3)

**Food Technology Neophobia** [25], based on [48]	The benefits of new food technologies are often grossly overstated.	Die Vorteile neuer Lebensmitteltechnologien werden oft maßlos überschätzt.	5-point Likert scale (1 = strongly disagree, 2 = disagree, 3 = partly, partly, 4 = agree, 5 = strongly agree)	5-point Likert scale (1 = stimme ganz und gar nicht zu, 2 = stimme nicht zu, 3 = teils, teils, 4 = stimme zu, 5 = stimme voll und ganz zu)	1 = 51 (7.8), 2 = 246 (37.9), 3 = 211 (32.5), 4 = 107 (16.5), 5 = 18 (2.8), NA = 4 (0.6)	all values were averaged into a mean score
	There are plenty of tasty foods around so we don't need to use new food technologies to produce more.	Es gibt genügend schmackhafte Lebensmittel, sodass wir keine neuen Lebensmitteltechnologien einsetzen müssen, um mehr zu produzieren.			1 = 130 (20.0), 2 = 249 (38.3), 3 = 159 (24.5), 4 = 78 (12.0), 5 = 17 (2.6), NA = 4 (0.6)	
	New food technologies decrease the natural quality of food.	Neue Lebensmitteltechnologien mindern die natürliche Qualität von Lebensmitteln.			1 = 106 (16.3), 2 = 264 (40.5), 3 = 200 (30.7), 4 = 48 (7.4), 5 = 15 (2.3), NA = 4 (0.6)	
**Food involvement** [49]	I just love good food.	Ich liebe einfach gutes Essen.	5-point Likert scale (1 = strongly disagree, 2 = disagree, 3 = partly, partly, 4 = agree, 5 = strongly agree)	5-point Likert scale (1 = stimme ganz und gar nicht zu, 2 = stimme nicht zu, 3 = teils, teils, 4 = stimme zu, 5 = stimme voll und ganz zu)	1 = 2 (0.3), 2 = 8 (1.2), 3 = 46 (7.1), 4 = 222 (34.8), 5 = 358 (56.1), NA = 1 (0.2)	all values were averaged into a mean score
	Eating and drinking are a continuous source of joy for me.	Essen und Trinken sind für mich eine ständige Quelle der Freude.			1 = 9 (1.4), 2 = 26 (4.0), 3 = 170 (26.2), 4 = 229 (35.3), 5 = 203 (31.3), NA = 0 (0)	
	Decisions on what to eat and drink are very important for me.	Die Entscheidung, was ich esse und trinke, ist für mich sehr wichtig.			1 = 4 (0.6), 2 = 11 (1.7), 3 = 109 (16.5), 4 = 249 (37.6), 5 = 264 (39.9), NA = 0 (0)	
	Food and drink is an important part of my life.	Essen und Trinken ist ein wichtiger Teil meines Lebens.			1 = 3 (0.5), 2 = 15 (2.4), 3 = 58 (8.8), 4 = 272 (41.4), 5 = 289 (44.0), NA = 0 (0)	
	Eating and food is an important part of my social life.	Essen und Trinken ist ein wichtiger Teil meines sozialen Lebens.			1 = 11 (1.7), 2 = 42 (6.6), 3 = 129 (20.3), 4 = 258 (40.5), 5 = 195 (30.6), NA = 2 (0.3)	
**Food innovativeness** [49]	Recipes and articles on food from other culinary traditions encourage me to	Rezepte und Berichte über Speisen aus anderen kulinarischen Traditionen regen mich	5-point Likert scale (1 = strongly disagree, 2 = disagree, 3 = partly,	5-point Likert scale (1 = stimme ganz und gar nicht zu, 2 = stimme nicht zu, 3 =	1 = 31 (4.8), 2 = 90 (13.9), 3 = 164 (25.4), 4 = 217	all values were averaged

	experiment in the kitchen.	zum Experimentieren in der Küche an.	partly, 4 = agree, 5 = strongly agree)	teils, teils, 4 = stimme zu, 5 = stimme voll und ganz zu)	(33.6), 5 = 135 (20.9), NA = 0 (0)	into a mean score
	I love to try recipes from different countries.	Ich liebe es, Rezepte aus anderen Ländern auszuprobieren.			1 = 17 (2.6), 2 = 54 (8.4), 3 = 175 (27.1), 4 = 209 (32.3), 5 = 182 (28.1), NA = 0 (0)	
	I like to try new foods that I have never tasted before.	Ich probiere gerne neue Lebensmittel aus, die ich noch nie probiert habe.			1 = 14 (2.2), 2 = 68 (10.6), 3 = 174 (27.0), 4 = 227 (35.2), 5 = 154 (23.9), NA = 0 (0)	
	I like to try out new recipes.	Ich probiere gerne neue Rezepte aus.			1 = 16 (2.5), 2 = 53 (8.1), 3 = 136 (20.9), 4 = 261 (40.1), 5 = 171 (26.3), NA = 0 (0)	
	I look for ways to prepare unusual meals.	Ich suche nach Möglichkeiten, außergewöhnliche Mahlzeiten zuzubereiten.			1 = 53 (8.3), 2 = 131 (20.5), 3 = 223 (34.9), 4 = 149 (23.3), 5 = 81 (12.7), NA = 0 (0)	
**Environmental consciousness** [47], based on [67]	When I buy food, I try to consider how my use of it will affect the environment.	Wenn ich Lebensmittel kaufe, versuche ich zu bedenken, welche Auswirkungen mein Konsum auf die Umwelt hat.	5-point Likert scale (1 = strongly disagree, 2 = disagree, 3 = partly, partly, 4 = agree, 5 = strongly agree)		1 = 24 (3.7), 2 = 63 (9.8), 3 = 188 (29.2), 4 = 245 (38.0), 5 = 117 (18.2), NA = 0 (0)	all values were averaged into a mean score
	It is important to me that the food was produced in an environmentally friendly way.	Es ist mir wichtig, dass die Lebensmittel auf umweltfreundliche Weise hergestellt wurden.			1 = 11 (1.7), 2 = 37 (5.7), 3 = 155 (23.9), 4 = 294 (45.4), 5 = 140 (21.6), NA = 0 (0)	
	It is important to me that the food has been packaged in an environmentally friendly way.	Es ist mir wichtig, dass die Lebensmittel umweltfreundlich verpackt sind.			1 = 19 (2.9), 2 = 31 (4.9), 3 = 154 (24.0), 4 = 303 (47.1), 5 = 130 (20.2), NA = 0 (0)	
	If given a choice, I choose the more environmentally friendly product, even at higher costs.	Wenn ich die Wahl habe, entscheide ich mich für das umweltfreundlichere Produkt, auch wenn es mehr kostet.			1 = 25 (3.9), 2 = 57 (8.9), 3 = 200 (31.4), 4 = 230 (36.1), 5 = 125	

					(19.6), NA = 0 (0)	
	I try to reduce my impact on the environment through the choice of food.	Ich versuche, die Umweltbelastung durch die Wahl der Lebensmittel zu reduzieren.			1 = 16 (2.5), 2 = 59 (9.2), 3 = 168 (26.1), 4 = 278 (43.2), 5 = 116 (18.0), NA = 0 (0)	
**Health consciousness** [25], based on [56]	The healthiness of food has little impact on my food choices. (R)	Die Gesundheit hat wenig Einfluss auf meine Lebensmittelauswahl. (R)			1 = 14 (2.2), 2 = 67 (10.5), 3 = 107 (16.7), 4 = 293 (45.6), 5 = 156 (24.3), NA = 0 (0)	all values were averaged into a mean score
	I am very particular about the healthiness of food I eat.	Ich bin sehr wählerisch, wenn es darum geht, wie gesund die Lebensmittel sind, die ich esse.	5-point Likert scale (1 = strongly disagree, 2 = disagree, 3 = partly, partly, 4 = agree, 5 = strongly agree)	5-point Likert scale (1 = stimme ganz und gar nicht zu, 2 = stimme nicht zu, 3 = teils, teils, 4 = stimme zu, 5 = stimme voll und ganz zu)	1 = 24 (3.9), 2 = 109 (17.7), 3 = 261 (42.3), 4 = 188 (30.4), 5 = 55 (8.9), NA = 0 (0)	
	I eat what I like and I do not worry much about the healthiness of food. (R)	Ich esse, was mir schmeckt, und mache mir keine großen Gedanken darüber, wie gesund die Lebensmittel sind. (R)			1 = 21 (3.3), 2 = 71 (11.5), 3 = 157 (25.5), 4 = 277 (45.1), 5 = 111 (18.1), NA = 0 (0)	
**Willingness to buy**[1] [89]	Would you buy algae/crickets/jellyfish as part of your regular diet in the future? (e.g. as a food product or as an ingredient in a food product)	Würdest Du Algen/Grillen/Quallen als Teil Deiner gewöhnlichen Ernährung in Zukunft kaufen? (z.B. als Lebensmittelprodukt oder als Inhaltsstoff in einem Lebensmittel)	no	nein	30 (4.7) / 187 (29.4) / 253 (39.7)	not willing to buy = 0
			rather no	eher nein	96 (15.1) / 223 (35.0) / 231 (36.3)	not willing to buy = 0
			rather yes	eher ja	244 (38.3) / 155 (24.3) / 117 (18.4)	willing to buy = 1
			yes	ja	264 (41.4) / 72 (11.3) / 31 (4.9)	willing to buy = 1
			missing value	3 (0.5) / 0 / 5 (0.8)	NA	

Note. The questionnaire was conducted in German language. (R) indicates reversed items. [1]Items were slightly modified. N= 637.

File S2: Item analyses

Table S2. Food neophobia.

Item	Cronbach's alpha	Cronbach's alpha if item is dropped	Item-total correlation	Item difficulty	Mean	Standard deviation	Minimum	Maximum
I am constantly sampling new and different foods. (R)	0.82	0.80	0.53	0.56	2.80	1.01	1	5
I don't trust new foods.	0.82	0.80	0.62	0.46	2.29	0.90	1	5

I like foods from different countries. (R)	0.82	0.81	0.52	0.36	1.81	0.78	1	5
If I don't know what is in a food, I won't try it.	0.82	0.82	0.42	0.62	3.10	1.10	1	5
At dinner parties, I will try a new food. (R)	0.82	0.79	0.63	0.44	2.19	0.98	1	5
Some food looks too weird to eat.	0.82	0.82	0.42	0.61	3.03	1.10	1	5
I am afraid to eat things I have never had before.	0.82	0.80	0.57	0.43	2.16	0.94	1	5
I am very particular about the foods I will eat.	0.82	0.81	0.49	0.62	3.10	1.09	1	5
I will eat almost anything. (R)	0.82	0.80	0.58	0.56	2.80	1.21	1	5

Table S3. Food technology neophobia.

Item	Cronbach's alpha	Cronbach's alpha if item is dropped	Item-total correlation	Item difficulty	Mean	Standard deviation	Minimum	Maximum
The benefits of new food technologies are often grossly overstated.	0.74	0.66	0.56	0.54	2.68	0.94	1	5
There are plenty of tasty foods around so we don't need to use new food technologies to produce more.	0.74	0.64	0.58	0.47	2.37	1.03	1	5
New food technologies decrease the natural quality of food.	0.74	0.66	0.56	0.47	2.37	0.93	1	5

Table S4. Food involvement.

Item	Cronbach's alpha	Cronbach's alpha if item is dropped	Item-total correlation	Item difficulty	Mean	Standard deviation	Minimum	Maximum
I just love good food.	0.8	0.75	0.63	0.89	4.46	0.71	1	5
Eating and drinking are a continuous source of joy for me.	0.8	0.74	0.67	0.79	3.93	0.93	1	5
Decisions on what to eat and drink are very important for me.	0.8	0.80	0.47	0.84	4.19	0.82	1	5
Food and drink is an important part of my life.	0.8	0.74	0.67	0.86	4.30	0.77	1	5
Eating and food is an important part of my social life.	0.8	0.79	0.53	0.78	3.92	0.96	1	5

Table S5. Food innovativeness.

Item	Cronbach's alpha	Cronbach's alpha if item is dropped	Item-total correlation	Item difficulty	Mean	Standard deviation	Minimum	Maximum
Recipes and articles on food from other culinary traditions encourage me to experiment in the kitchen.	0.9	0.87	0.79	0.71	3.53	1.12	1	5
I love to try recipes from different countries.	0.9	0.87	0.81	0.75	3.76	1.04	1	5
I like to try new foods that I have never tasted before.	0.9	0.90	0.64	0.74	3.69	1.02	1	5
I like to try out new recipes.	0.9	0.88	0.76	0.76	3.81	1.00	1	5
I look for ways to prepare unusual meals.	0.9	0.87	0.78	0.62	3.12	1.13	1	5

Table S6. Environmental consciousness.

Item	Cronbach's alpha	Cronbach's alpha if item is dropped	Item-total correlation	Item difficulty	Mean	Standard deviation	Minimum	Maximum
When I buy food, I try to consider how my use of it will affect the environment.	0.89	0.86	0.77	0.72	3.58	1.02	1	5
It is important to me that the food was produced in an environmentally friendly way.	0.89	0.86	0.75	0.76	3.81	0.90	1	5
It is important to me that the food has been packaged in an environmentally friendly way.	0.89	0.87	0.69	0.76	3.78	0.93	1	5
If given a choice, I choose the more environmentally friendly product, even at higher costs.	0.89	0.88	0.66	0.72	3.59	1.03	1	5
I try to reduce my impact on the environment through the choice of food.	0.89	0.85	0.79	0.73	3.66	0.96	1	5

Table S7. Health consciousness.

Item	Cronbach's alpha	Cronbach's alpha if item is dropped	Item-total correlation	Item difficulty	Mean	Standard deviation	Minimum	Maximum
The healthiness of food has little impact on my food choices. (R)	0.69	0.62	0.49	0.76	3.80	1.00	1	5
I am very particular about the healthiness of food I eat.	0.69	0.66	0.46	0.64	3.22	0.96	1	5
I eat what I like and I do not worry much about the healthiness of food. (R)	0.69	0.51	0.58	0.72	3.61	1.00	1	5

File S3: Comparison between German population and sample

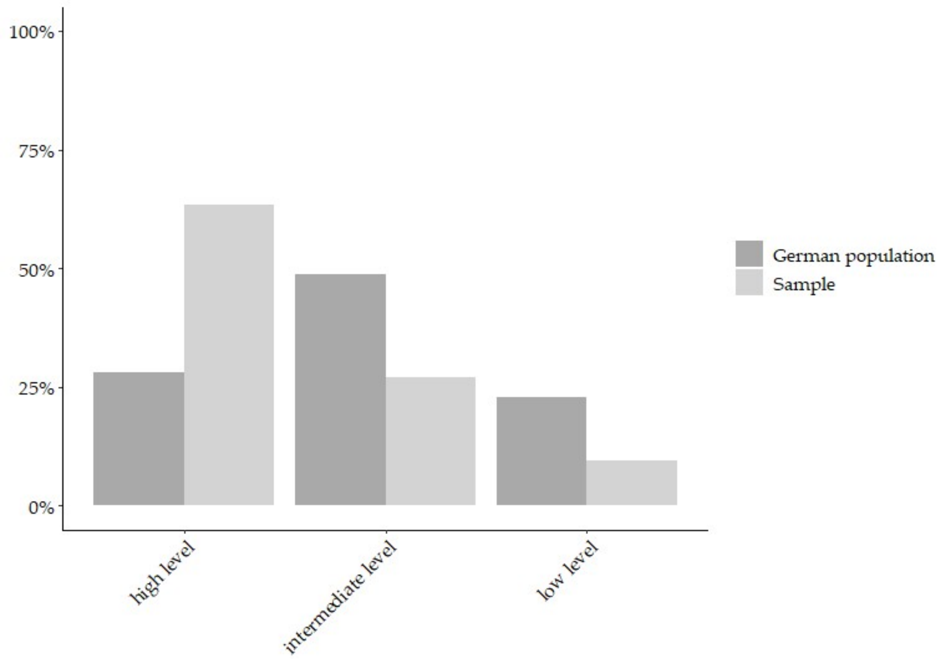

Figure S1. Comparison of Education Distribution: German Population vs. Sample. Taken from [60].

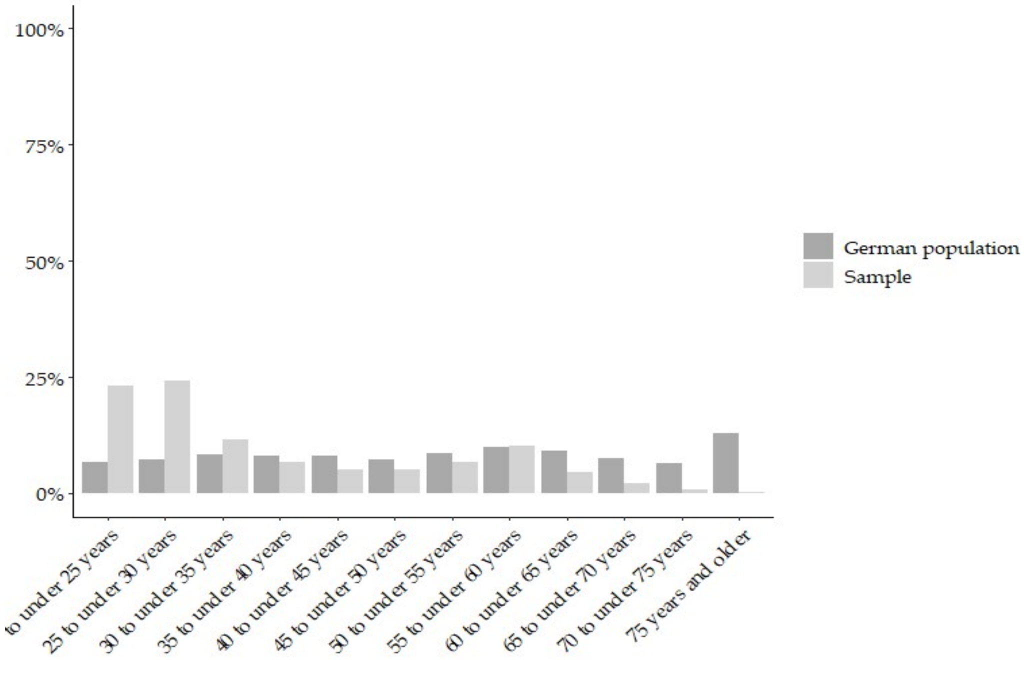

Figure S2. Comparison of Age Distribution: German Population vs. Sample. Taken from [83].

File S4: Predicted probability curves

In the following, all predicted probability curves for each predictor included in the final models are presented for the three alternative protein sources, algae, crickets and jellyfish, excluding

those already shown in the main text (environmental consciousness for algae acceptance, meat consumption for cricket acceptance and food innovativeness for jellyfish acceptance). The predicted probability curves with 95% confidence intervals are derived using the ‘ggpredict()’ function from the ‘ggeffects’ package in R from Lüdecke (v2.3.0) [84], while holding all other variables at their mean or reference levels.

Algae

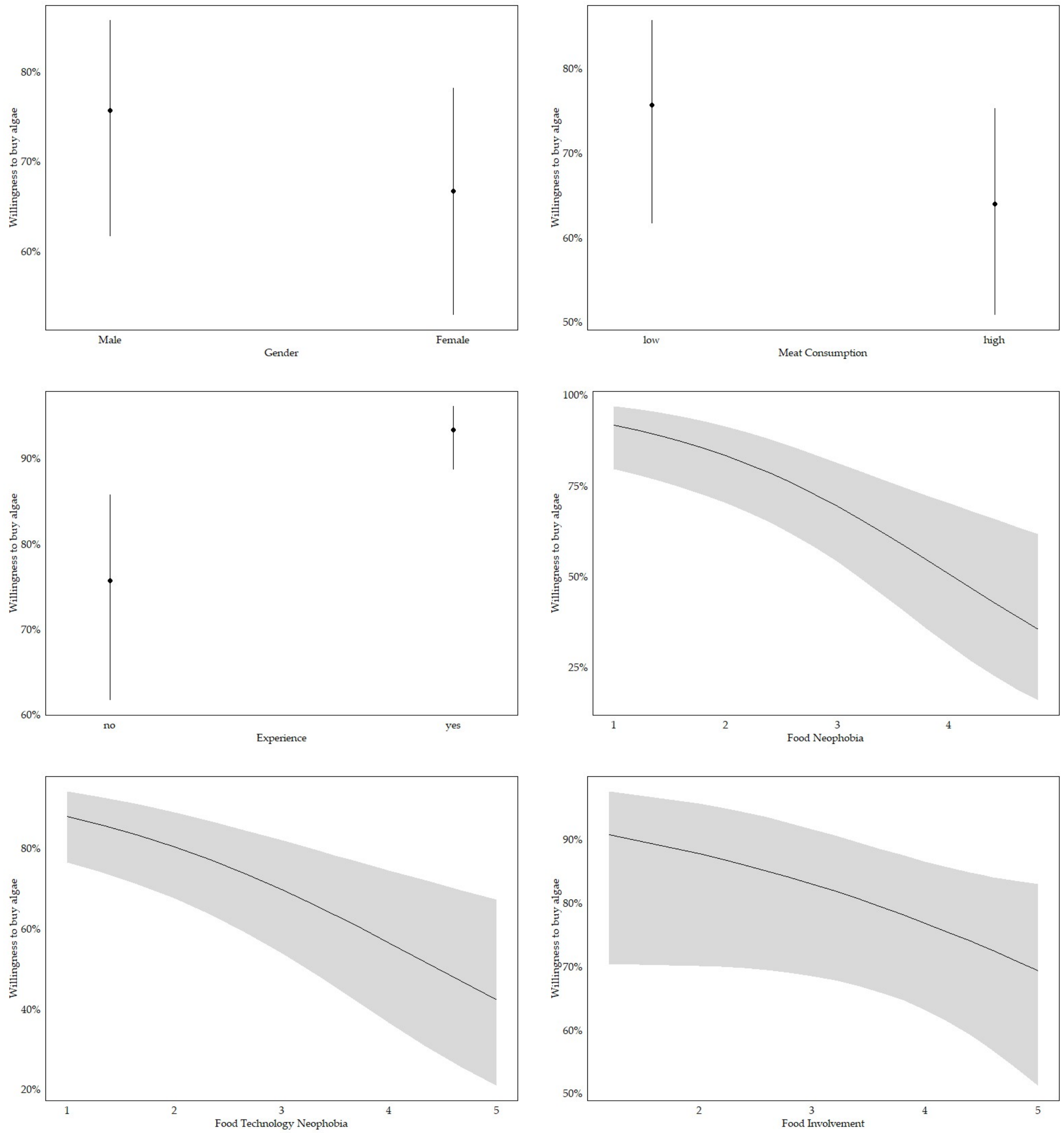

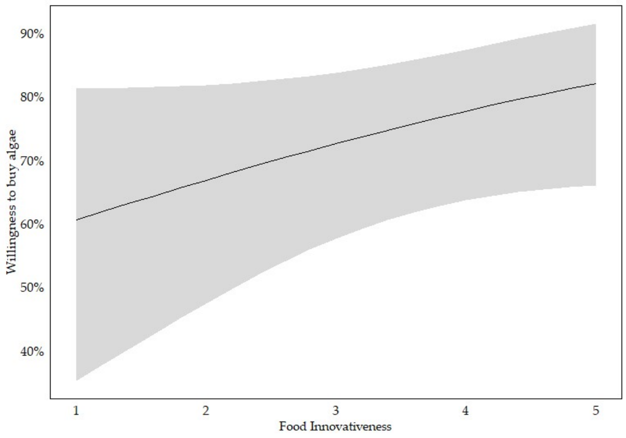
90%
80%
70%
60%
50%
40%
Willingness to buy algae
1
2
3
4
5
Food Innovativeness

Crickets

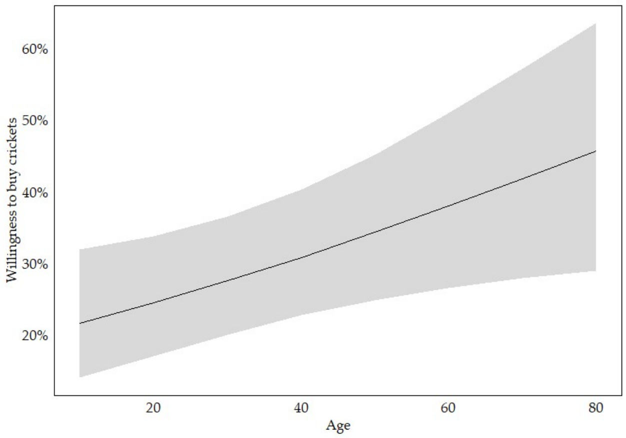
60%
50%
40%
30%
20%
Willingness to buy crickets
20
40
60
80
Age

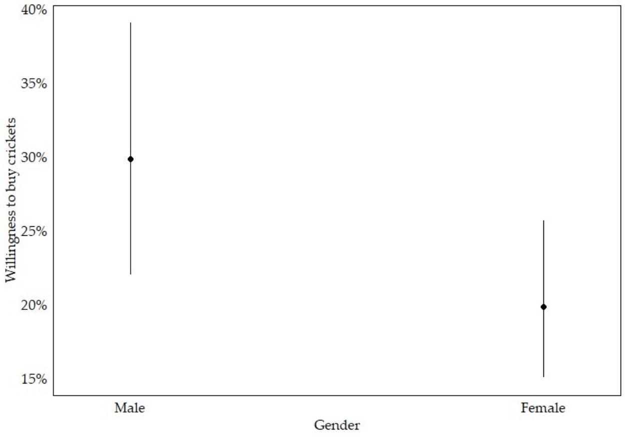
40%
35%
30%
25%
20%
15%
Willingness to buy crickets
Male
Female
Gender

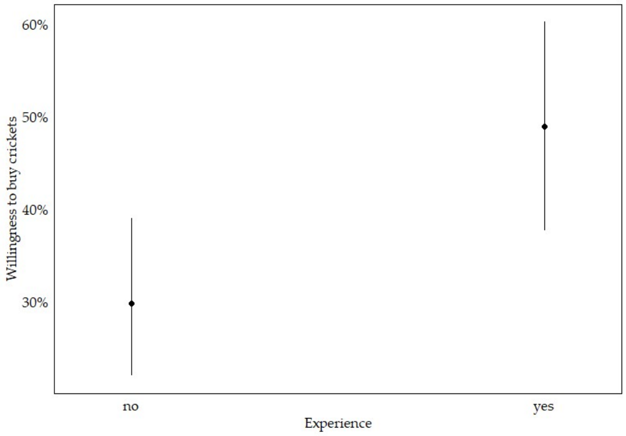
60%
50%
40%
30%
Willingness to buy crickets
no
yes
Experience

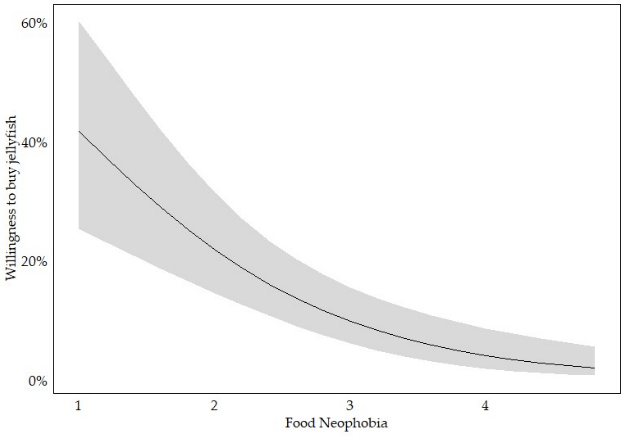
60%
40%
20%
0%
Willingness to buy jellyfish
1
2
3
4
Food Neophobia

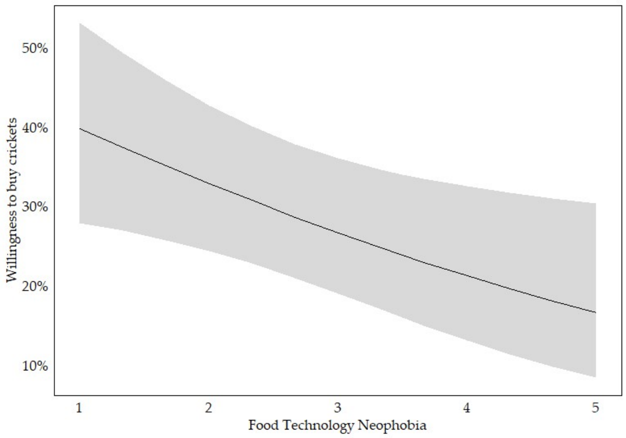

Willingness to buy crickets
50%
40%
30%
20%
10%
1
2
3
4
5
Food Technology Neophobia

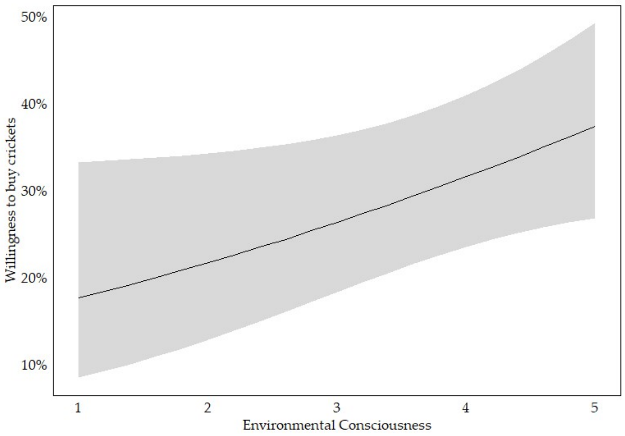

Willingness to buy crickets
50%
40%
30%
20%
10%
1
2
3
4
5
Environmental Consciousness

Jellyfish

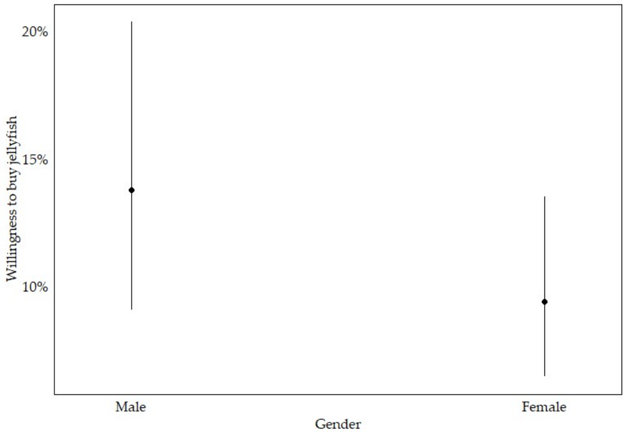

Willingness to buy jellyfish
20%
15%
10%
Male
Female
Gender

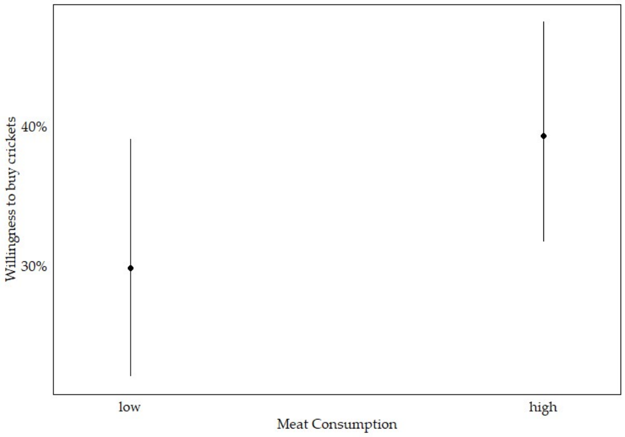

Willingness to buy crickets
40%
30%
low
high
Meat Consumption

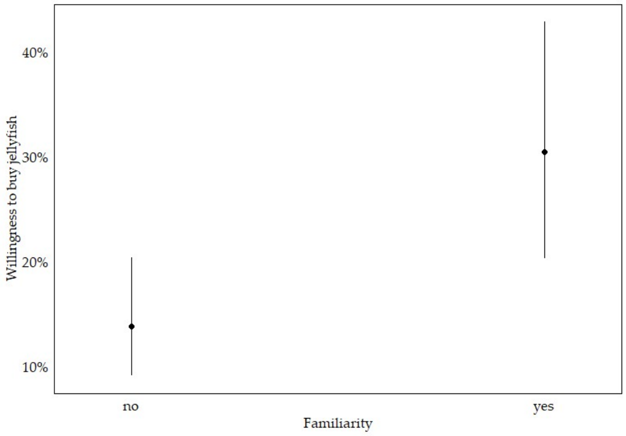

Willingness to buy jellyfish
40%
30%
20%
10%
no
yes
Familiarity

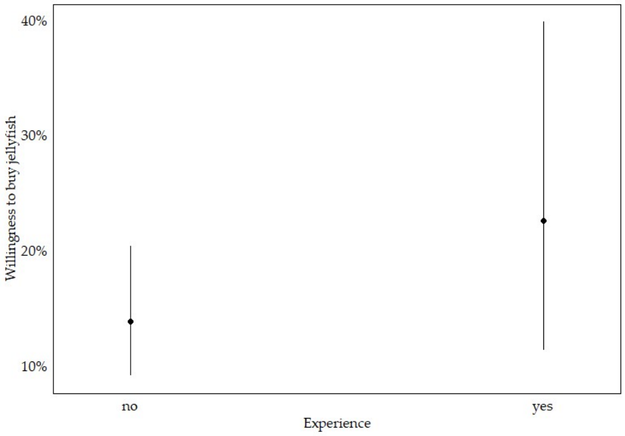

Willingness to buy jellyfish
40%
30%
20%
10%
no
yes
Experience

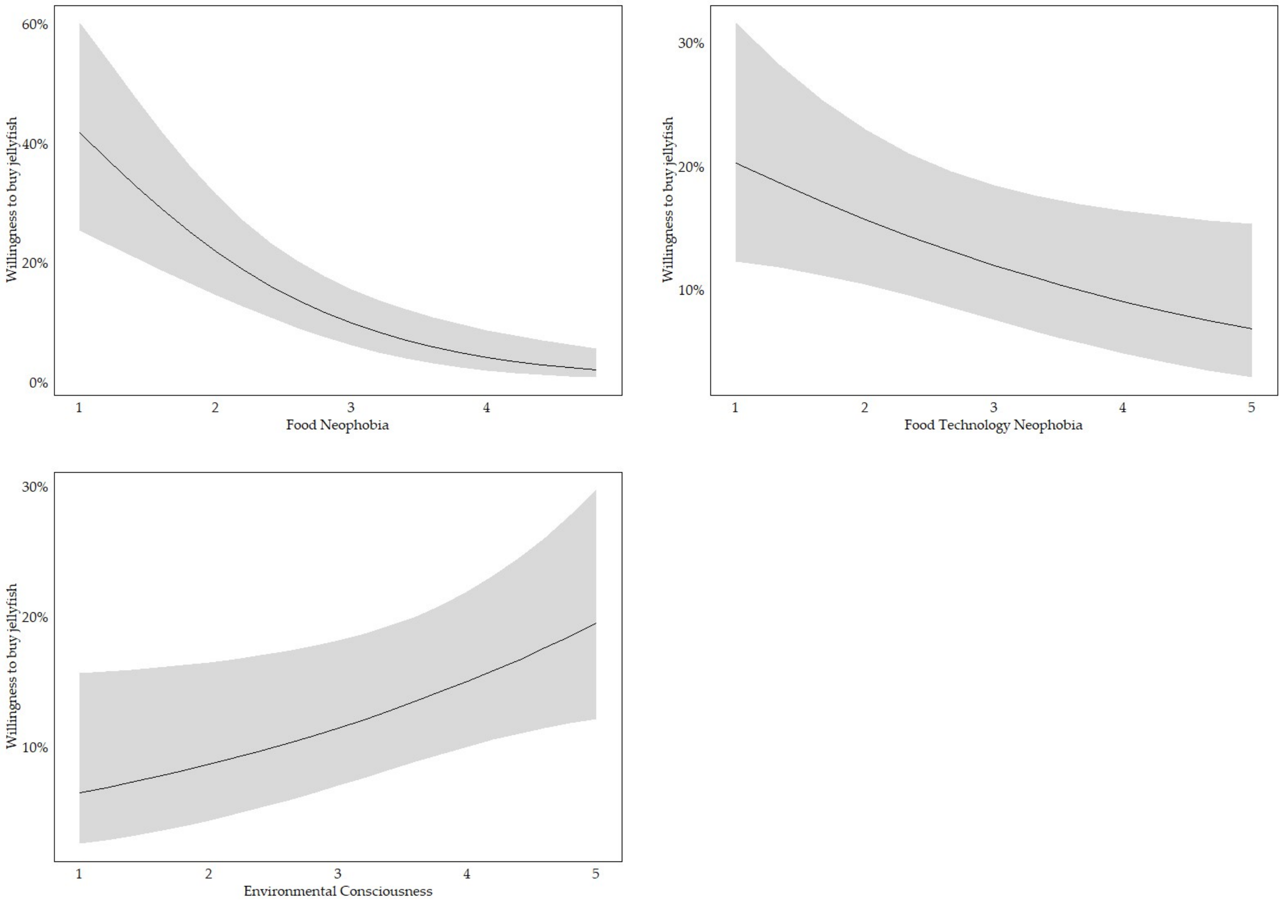
60%
40%
20%
0%
Willingness to buy jellyfish
1
2
3
4
Food Neophobia
30%
20%
10%
Willingness to buy jellyfish
1
2
3
4
5
Food Technology Neophobia
30%
20%
10%
Willingness to buy jellyfish
1
2
3
4
5
Environmental Consciousness

File S5: Stepwise logistic regression

All analyses were performed using R statistical software (v4.2.1 [68]). To investigate the factors influencing respondents' willingness to buy food products containing the respective alternative protein source, a binary logistic regression model was employed. The selection of the final model's parameters was determined through stepwise regression, guided by the 'Akaike Information Criterion' (AIC) using the 'MASS' package (stepAIC(full.model, direction = "both") in R (v7.3.57 [69]).

Note: Meat consumption (MeatCons), familiarity (Fam), experience (Exp), food neophobia (FoodNeo), food technology neophobia (FoodTechNeo), food involvement (FoodInv), food innovativeness (FoodInno), environmental consciousness (Env).

Algae

Start: AIC=500.92

Willingness_to_buy ~ Age + Gender + Edu + MeatCons + Fam + Exp + FoodNeo + FoodTechNeo + FoodInv + FoodInno + Env

	AIC
- Edu	499.15
- Age	499.31
- Fam	499.78
<none>	500.92
- FoodInno	501.83
- FoodInv	502.03
- Gender	502.24
- MeatCons	504.23
- Env	510.45
- FoodNeo	511.95
- FoodTechNeo	512.62
- Exp	524.91

Step: AIC=499.15

Willingness_to_buy ~ Age + Gender + MeatCons + Fam + Exp + FoodNeo + FoodTechNeo + FoodInv + FoodInno + Env

	AIC
- Age	497.55

- Fam	497.95
<none>	499.15
- FoodInno	499.99
- FoodInv	500.23
- Gender	500.38
+ Edu	500.92
- MeatCons	502.56
- Env	508.46
- FoodNeo	510.16
- FoodTechNeo	510.77
- Exp	523.07

Step: AIC=497.55

Willingness_to_buy ~ Gender + MeatCons + Fam + Exp + FoodNeo + FoodTechNeo + FoodInv + FoodInno + Env

	AIC
- Fam	496.30
<none>	497.55
- FoodInno	498.32
- Gender	498.67
- FoodInv	498.70
+ Age	499.15
+ Edu	499.31
- MeatCons	500.71
- FoodNeo	508.29
- Env	508.54
- FoodTechNeo	508.82
- Exp	522.08

Step: AIC=496.3

Willingness_to_buy ~ Gender + MeatCons + Exp + FoodNeo + FoodTechNeo + FoodInv + FoodInno + Env

	AIC
<none>	496.30
- FoodInno	496.80
- Gender	497.34
- FoodInv	497.43
+ Fam	497.55
+ Age	497.95
+ Edu	498.11
- MeatCons	499.09
- FoodNeo	507.34
- FoodTechNeo	507.77
- Env	507.77
- Exp	524.95

	VIF(full.model)	VIF(step.model)
Age	1.101384	
Gender	1.131147	1.123373
Edu	1.027319	
MeatCons	1.212288	1.180553
Fam	1.096275	
Exp	1.137734	1.063364
FoodNeo	1.441769	1.436866
FoodTechNeo	1.103166	1.086792
FoodInv	1.417727	1.408232

FoodInno	1.769646	1.749890
Env	1.198994	1.132154

Crickets

Start: AIC=500.92 Start: AIC=708.25

Willingness_to_buy ~ Age + Gender + Edu + MeatCons + Fam + Exp + FoodNeo + FoodTechNeo + FoodInv + FoodInno + Env

	AIC
- Fam	706.27
- FoodInv	706.28
- FoodInno	706.72
- Edu	707.06
<none>	708.25
- MeatCons	710.16
- Env	710.23
- FoodTechNeo	711.19
- Age	711.95
- Gender	713.66
- Exp	721.90
- FoodNeo	750.75

Step: AIC=706.27

Willingness_to_buy ~ Age + Gender + Edu + MeatCons + FoodNeo + FoodTechNeo + FoodInv + FoodInno + Env

	AIC
- FoodInv	704.30
- FoodInno	704.75
- Edu	705.07
<none>	706.27

- MeatCons	708.18
- Env	708.23
+ Fam	708.25
- FoodTechNeo	709.19
- Age	709.97
- Gender	711.66
- Exp	719.93
- FoodNeo	748.76

Step: AIC=704.3

Willingness_to_buy ~ Age + Gender + Edu + MeatCons + Exp + FoodNeo + FoodTechNeo + FoodInno + Env

	AIC
- FoodInno	703.00
- Edu	703.08
<none>	704.30
- MeatCons	706.18
- Env	706.23
+ FoodInv	706.27
+ Fam	706.28
- FoodTechNeo	707.24
- Age	708.06
- Gender	709.85
- Exp	718.03
- FoodNeo	746.86

Step: AIC=703

Willingness_to_buy ~ Age + Gender + Edu + MeatCons + Exp + FoodNeo + FoodTechNeo + Env

	AIC
- Edu	701.70
<none>	703.00
+ FoodInno	704.30
- Env	704.51
+ FoodInv	704.75
+ Fam	704.96
- MeatCons	705.02
- FoodTechNeo	706.27
- Age	706.68
- Gender	708.86
- Exp	716.56
- FoodNeo	751.48

Step: AIC=701.7

Willingness_to_buy ~ Age + Gender + MeatCons + Exp + FoodNeo + FoodTechNeo + Env

	AIC
<none>	701.70
+ Edu	703.00
+ FoodInno	703.08
+ FoodInv	703.51
+ Fam	703.68
- Env	703.74
- MeatCons	703.98
- FoodTechNeo	705.14
- Age	705.22
- Gender	707.57

- Exp	715.76
- FoodNeo	751.08

	VIF(full.model)	VIF(step.model)
Age	1.106096	1.098296
Gender	1.086962	1.069253
Edud	1.045887	
MeatCons	1.212031	1.198742
Fam	1.036964	
Exp	1.049874	1.036968
FoodNeo	1.369606	1.104224
FoodTechNeo	1.084691	1.074446
FoodInv	1.297965	
FoodInno	1.578180	
Env	1.237520	1.170252

Jellyfish

Start: AIC=583.53

Willingness_to_buy ~ Age + Gender + Edu + MeatCons + Fam + Exp + FoodNeo + FoodTechNeo + FoodInv + FoodInno + Env

	AIC
- Edu	581.53
- FoodInv	582.06
- Age	582.83
<none>	583.53
- Exp	584.11
- Env	585.16
- Gender	585.20

- MeatCons 586.15

- FoodTechNeo 586.38

- FoodInno 587.10

- Fam 599.96

- FoodNeo 603.60

Step: AIC=581.53

Willingness_to_buy ~ Age + Gender + MeatCons + Fam + Exp + FoodNeo + FoodTechNeo + FoodInv + FoodInno + Env

	AIC
- FoodInv	580.07
- Age	580.83
<none>	581.53
- Exp	582.12
- Gender	583.21
- Env	583.21
+ Edu	583.53
- MeatCons	584.17
- FoodTechNeo	584.41
- FoodInno	585.10
- Fam	598.00
- FoodNeo	601.68

Step: AIC=580.07

Willingness_to_buy ~ Age + Gender + Edu + MeatCons + Fam + Exp + FoodNeo + FoodTechNeo + FoodInno + Env

	AIC
- Age	579.48

<none>	580.07
- Exp	580.60
+ FoodInv	581.53
- Env	581.53
+ Edu	582.06
- Gender	582.15
- MeatCons	582.47
- FoodTechNeo	582.97
- FoodInno	583.15
- Fam	596.40
- FoodNeo	600.47

Step: AIC=579.48

Willingness_to_buy ~ Gender + MeatCons + Fam + Exp + FoodNeo + FoodTechNeo + FoodInno + Env

	AIC
<none>	579.48
+ Age	580.07
- Exp	580.13
+ FoodInv	580.83
- Gender	581.40
+ Edu	581.47
- Env	581.94
- FoodTechNeo	582.08
- FoodInno	582.51
- MeatCons	582.57
- Fam	597.69
- FoodNeo	598.81

	VIF(full.model)	VIF(step.model)
Age	1.136561	
Gender	1.068748	1.054624
Edu	1.053098	
MeatCons	1.203638	1.177496
Fam	1.106630	1.086422
Exp	1.073078	1.067949
FoodNeo	1.293819	1.252482
FoodTechNeo	1.115888	1.105913
FoodInv	1.271413	
FoodInno	1.433346	1.208192
Env	1.223616	1.158589

References:

25. Verbeke, W. Profiling consumers who are ready to adopt insects as a meat substitute in a Western society. *Food Qual. Prefer.* **2015**, *39*, 147–155. https://doi.org/10.1016/j.foodqual.2014.07.008.
41. Pliner, P.; Hobden, K. Development of a scale to measure the trait of food neophobia in humans. *Appetite* **1992**, *19*, 105–120. https://doi.org/10.1016/0195-6663(92)90014-W.
47. Orsi, L.; Voege, L.L.; Stranieri, S. Eating edible insects as sustainable food? Exploring the determinants of consumer acceptance in Germany. *Food Res. Int.* **2019**, *125*, 108573. https://doi.org/10.1016/j.foodres.2019.108573.
48. Cox, D.N.; Evans, G. Construction and validation of a psychometric scale to measure consumers' fears of novel food technologies: The food technology neophobia scale. *Food Qual. Prefer.* **2008**, *19*, 704–710. https://doi.org/10.1016/j.foodqual.2008.04.005.
49. Brunsø, K.; Birch, D.; Memery, J.; Temesi, Á.; Lakner, Z.; Lang, M.; Dean, D.; Grunert, K.G. Core dimensions of food-related lifestyle: A new instrument for measuring food involvement, innovativeness and responsibility. *Food Qual. Prefer.* **2021**, *91*, 104192. https://doi.org/10.1016/j.foodqual.2021.104192.
56. Roininen, K.; Lähteenmäki, L.; Tuorila, H. Quantification of consumer attitudes to health and hedonic characteristics of foods. *Appetite* **1999**, *33*, 71–88. https://doi.org/10.1006/appe.1999.0232.
60. Federal Statistical Office of Germany. Graduates and Leavers: Germany, School Year, Gender, Type of School, School-Leaving Qualifications (Table 21111-0004). Available online: https://www.govdata.de/dl-de/by-2-0 (accessed on 12 September 2024).
61. Lea, E.; Worsley, A. Influences on meat consumption in Australia. *Appetite* **2001**, *36*, 127–136. https://doi.org/10.1006/appe.2000.0386.
62. Neves, Ane Telles Sposito Gonçalves. Determinants of Consumers' Acceptance of Insects as Food and Feed: A Cross-Cultural Study. 2015. Available online: https://core.ac.uk/download/pdf/143407494.pdf (accessed on 12 September 2024).

67. Meixner, O.; Mörl von Pfalzen, L. *Die Akzeptanz von Insekten in der Ernährung: Eine Studie zur Vermarktung von Insekten als Lebensmittel aus Konsumentensicht*; Springer Gabler: Wiesbaden/Heidelberg, Germany, 2018; ISBN 9783658213350.
68. R Core Team. *R: A Language and Environment for Statistical Computing*; R Core Team: Vienna, Austria, 2021. Available online: https://www.R-project.org/ (accessed on 12 September 2024).
69. Venables, W.N.; Ripley, B.D. *Modern Applied Statistics with S.*; Springer: New York, NY, USA, 2002; ISBN 0-387-95457-0.
83. Federal Statistical Office of Germany. Population Aged 15 and over in Main Residence Households: Germany, Years, Gender, Age Groups, Educational Status (Table 12211-0102). Available online: www.govdata.de/dl-de/by-2-0 (accessed on 12 September 2024).
84. Lüdecke, D. Ggeffects: Tidy Data Frames of Marginal Effects from Regression Models. *Journal of Open Source Software* **2018**, 3, 26, 772. https://doi.org/10.21105/joss.00772.
89. Tan, H.S.G.; Verbaan, Y.T.; Stieger, M. How will better products improve the sensory-liking and willingness to buy insect-based foods? *Food Res. Int.* **2017**, *92*, 95–105. https://doi.org/10.1016/j.foodres.2016.12.021.
